# Graphene-based nanomaterials for breast cancer treatment: promising therapeutic strategies

**DOI:** 10.1186/s12951-021-00902-8

**Published:** 2021-07-15

**Authors:** Guangman Cui, Junrong Wu, Jiaying Lin, Wenjing Liu, Peixian Chen, Meng Yu, Dan Zhou, Guangyu Yao

**Affiliations:** 1 Breast Center, Department of General Surgery, Nanfang Hospital, Southern Medical University, Guangzhou, China; 2 Stomatological Hospital, Southern Medical University, Guangzhou, China; 3 Department of Breast Surgery, The First People’s Hospital of Foshan, Sun Yat-Sen University, Guangdong, China; 4 Guangdong Provincial Key Laboratory of New Drug Screening, School of Pharmaceutical Sciences, Southern Medical University, Guangzhou, China

**Keywords:** Graphene-based nanomaterial, Breast cancer, Therapeutic strategies, Targeting

## Abstract

Breast cancer is the most common malignancy in women, and its incidence increases annually. Traditional therapies have several side effects, leading to the urgent need to explore new smart drug-delivery systems and find new therapeutic strategies. Graphene-based nanomaterials (GBNs) are potential drug carriers due to their target selectivity, easy functionalization, chemosensitization and high drug-loading capacity. Previous studies have revealed that GBNs play an important role in fighting breast cancer. Here, we have summarized the superior properties of GBNs and modifications to shape GBNs for improved function. Then, we focus on the applications of GBNs in breast cancer treatment, including drug delivery, gene therapy, phototherapy, and magnetothermal therapy (MTT), and as a platform to combine multiple therapies. Their advantages in enhancing therapeutic effects, reducing the toxicity of chemotherapeutic drugs, overcoming multidrug resistance (MDR) and inhibiting tumor metastasis are highlighted. This review aims to help evaluate GBNs as therapeutic strategies and provide additional novel ideas for their application in breast cancer therapy.

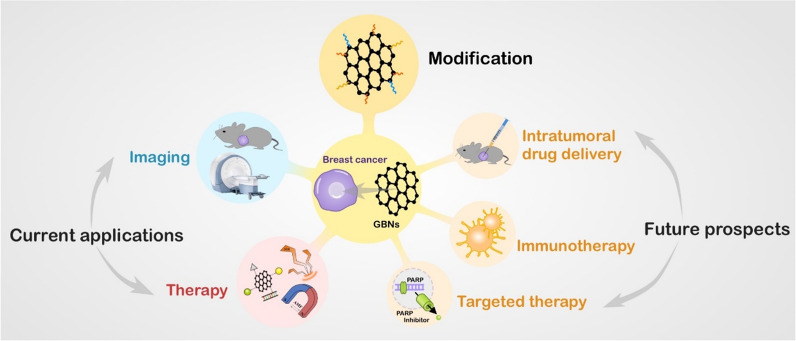

## Introduction

On December 15, 2020, the International Agency for Research on Cancer of the World Health Organization released global cancer burden data on its official website, noting the 2.26 million new cases of breast cancer in the world and that breast cancer had officially replaced lung cancer as the world's most common cancer [1]. Although the survival rate of breast cancer patients is higher than that of patients with other solid tumors, the high incidence, younger age of onset, aggressiveness, early lymph node metastasis and poor prognosis of breast cancer, especially triple-negative breast cancer, cannot be ignored [2]. Due to the heterogeneity of breast cancer and complexity of tumor regulation mechanisms, biomedical treatment strategies face great challenges in the clinic. Currently, the main treatment strategies for breast cancer include surgery, chemotherapy, radiotherapy, immunotherapy, and hormone therapy, which may not completely kill the tumor and may produce adverse side effects [3]. Innovative new therapies are still needed, especially for patients with poorly treated breast cancer, such as those with triple-negative breast cancer and metastatic breast cancer. The development of nanomedicines has brought new perspectives to the treatment of cancer [4]. Among the several kinds of nanoparticles (NPs), graphene-based nanomaterials (GBNs) stand out due to their unique chemical structures. As 2D nanomaterials, GBNs have the unique advantages of an ultrathin structure, a large surface area, good electrical conductivity, excellent optical performance, good mechanical properties and so on, so both pristine and modified GBNs have drawn research interest from a wide range of fields [5].

Graphene, a single sheet of sp^2^ bonded carbon atoms, has a large surface area of approximately 2630 m^2^/g, and the surface groups of GBNs are easy to modify, giving them the advantages of a high drug-loading capacity, targeting specificity, and intelligent controlled release patterns [6, 7]. GBNs, especially modified GBNs, present low toxicity to normal cells at conventional concentrations, which illustrates their good biological safety [8, 9]. In addition, GBNs have excellent optical properties. They can absorb the near-infrared (NIR) spectrum at a wavelength of 650–900 nm, which penetrates to a depth of approximately 4–10 cm, and locally convert light into heat [10]. Additionally, GBNs have great conductivity and thermal conductivity, strong mechanical strength and atomic-scale tunability [11]. Unlike other carbon nanomaterials, GBNs can produce a half-integer quantum Hall effect on electrons and holes even at room temperature [12]. Moreover, GBNs have the advantages of low cost and large-scale preparation. Because of these attractive characteristics, GBNs are widely used in biomedical fields; therefore, GBNs are expected to bring new methods for the diagnosis and treatment of breast cancer.

In this review, we discuss the dilemmas in breast cancer treatment and latest advances in GBNs to overcome these challenges. We highlight the superior properties of GBNs, specifically focusing on the modification of GBNs and development of GBNs for the diagnosis and imaging of breast tumors, delivery of chemotherapeutic drugs and genes, phototherapy, MTT and combination therapy for the treatment of breast cancer. In addition, we discuss future development trends in the utilization of graphene-based intratumoral drug delivery as well as developing GBNs into targeted therapy and immunotherapy to block tumor growth through specific interference and promote breast cancer treatment. This review mainly focuses on all published information on GBNs related to breast cancer to date, summarizing the unique characteristics of GBNs and their application in breast cancer. Thus, this paper will be of interest to breast surgeons, drug developers, materials scientists, etc.

### Properties of GBNs for cancer treatment

**Fig. 1:**
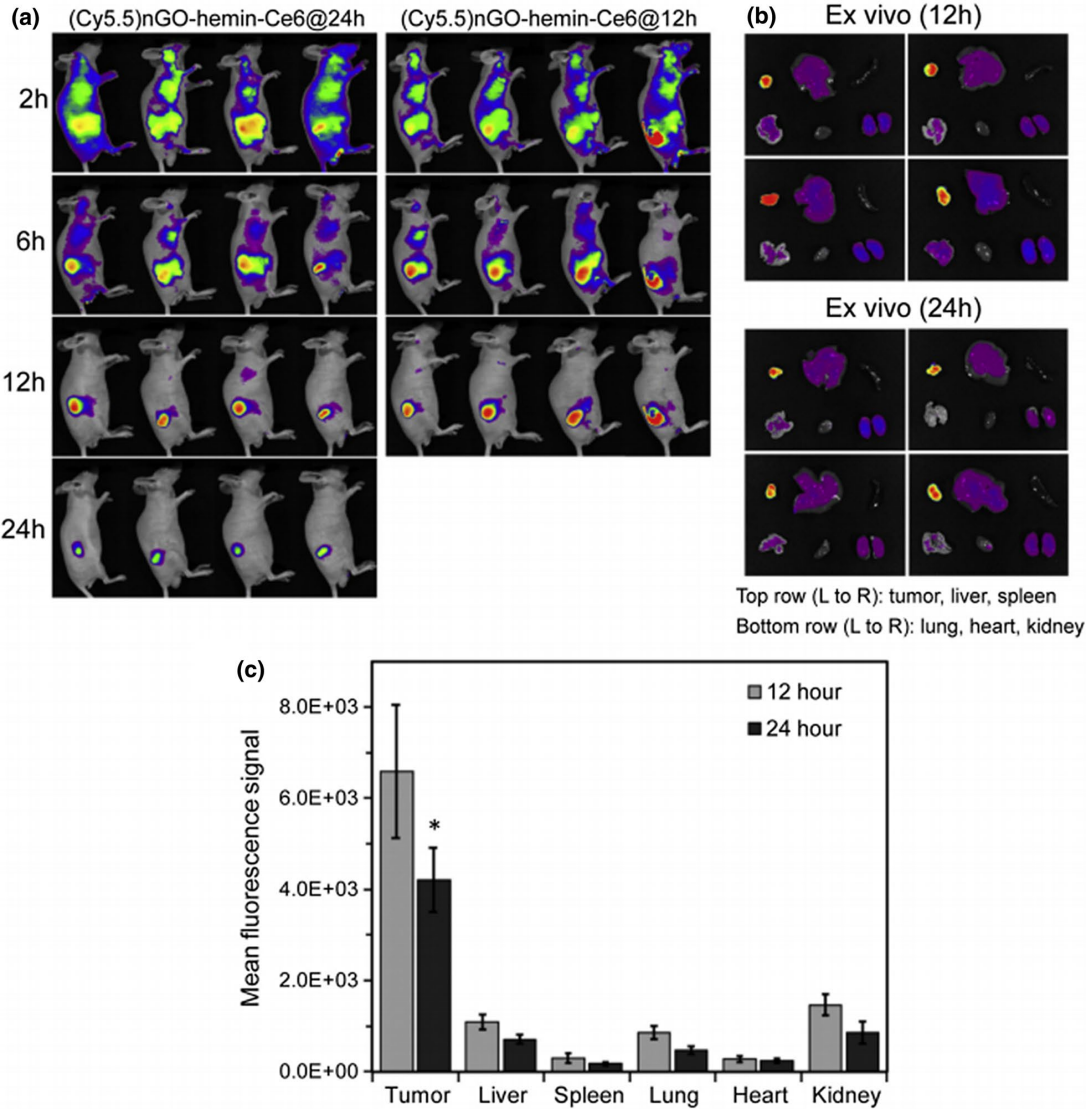
In vivo biodistribution of a GO-based nanosystem in a mouse tumor xenograft model. **a** Fluorescence imaging of tumor-bearing mice after intravenous injection of a fluorescence-labeled GO-based nanosystem at different times. **b** The GO-based nanosystem mainly accumulated in tumors, which showed the highest fluorescence intensity. **c** Mean fluorescence signal indicating the GO-based nanosystem distribution in tumors and other major organs [18]. Reprinted from Journal of Controlled Release, 326, Sahu A, Min K, Jeon J, Yang HS, Tae G, Catalytic nanographene oxide with hemin for enhanced photodynamic therapy, 442–454, 2020, with permission from Elsevier

Over the past few decades, GBNs have been rapidly developed and widely used. With increasing research on GBNs and the increase in their application, the metabolism, distribution, clearance, and other characteristics of GBNs in vivo and in vitro have received increasing attention. Many studies have evaluated the interaction between GBNs and various life systems, such as microorganisms, mammalian cells and animal models [13]. GBNs were found to be distributed throughout the whole mouse body and can be gradually degraded by enzymatic oxidation by horseradish peroxidase [14]. Intravenously administered GBNs were reported to be concentrated in reticuloendothelial systems such as the liver and spleen [15, 16]. The current view is that GBNs can be removed from the body over time, possibly through renal clearance or excretion by the biliary tract [16]. The size and modification of GBNs are important factors that affect their distribution and metabolism. Because of their small size, graphene quantum dots (GQDs) have fast renal-clearance and biodegradation rates [17]. GBNs used in experiments are usually modified and can also be distributed throughout the body, but they target tumor tissue and mostly accumulate in tumor tissue. For example, Sahu et al. evaluated the in vivo biodistribution of a graphene oxide (GO)-based system (nGO-heminCe6) in a mouse tumor xenograft model, and the results showed that the whole tumor exhibited the highest fluorescence intensity, followed by the fluorescence intensity of the kidney, liver and lung, while the spleen and heart showed very low signal (Fig. 1) [18]. In in vitro experiments, GO was mainly distributed in the cytoplasm, mitochondria, endoplasmic reticulum and nucleus, while GQDs were mainly distributed in the cytoplasm [19, 20]. After GBN exposure, the important organs of the mice were stained with hematoxylin–eosin. The results showed no obvious histopathological abnormalities [18]. Thus, GBNs were confirmed to have good biological safety for application in cancer diagnosis and treatment.

Graphene and its derivatives are widely used in the biomedical field due to their high surface area; simple surface functionalization; and unique biological, electrical, thermal and mechanical properties [21]. These properties make GBNs attractive, especially for breast cancer treatment. GBNs have intrinsic anticancer properties and can enhance cell adhesion and capture breast cancer cells [22]. The toxic effects of graphene on tumor cells may occur through oxidative stress and autophagy [23]. Studies have shown that GBNs can reduce the activity of macrophages, resulting in oxidative damage. Moreover, they can inhibit the migration and invasion of breast cancer cells and inhibit tumor growth and metastasis by inhibiting mitochondrial respiration [22]. After exposure to GBNs, the permeability of the tumor cell membrane increases, which is conducive to drug delivery [24]. In addition, GBNs can activate the immune system by inducing the maturation of dendritic cells to promote antitumor immunity, creating opportunities for immunotherapy. Due to these properties, GBNs have been applied for breast cancer treatment. GBNs comprise different members with various chemicophysical properties, and their application in breast cancer slightly differs. The materials for breast cancer treatment that we have reviewed herein include pristine graphene, GO, reduced graphene oxide (rGO) and GQDs. Unlike other GBNs, pristine graphene has been used in only a few studies because of its poor solubility, dispersion in water and physiologically relevant conditions. Thus, we mainly discuss the properties of GO, rGO and GQDs for cancer treatment.

GO is an oxide of graphene that is usually prepared by the Hummers method. GO can be regarded as a nontraditional soft material with the characteristics of polymer, colloid, film and amphoteric molecules. The abundant alcohol, carboxyl acid, and epoxide functional groups in GO endow it with good water dispersibility [25]. These groups are easy to modify and form covalent bonds. Thus, GO is easily modified to increase its hydrophilicity and reduce its thickness when converted into its oxidized form. However, it is necessary to properly interface with biological systems through size control or size separation on various length scales. GO has great advantages as a drug carrier because it can adsorb a large number of drugs via π–π stacking because of its large specific surface area [26]. The tumor-killing efficiency of anticancer drugs is improved, and their dosage can be reduced when these drugs are bound to GO; furthermore, they show smart drug-release properties when bound to GO [8]. In addition, GO itself, especially in nanosheets with a size of approximately 100–200 nm, has anticancer properties, which have been shown to inhibit breast tumor growth and breast cancer cell metastasis [27]. This effect may be attributed to the downregulated expression of energy metabolism-related genes, decreased mitochondrial oxidative phosphorylation, and inhibition of ATP synthesis, resulting in the disruption of F-actin cytoskeleton assembly, which affects cell migration. Moreover, GO does not show obvious toxicity to normal cells [28].

rGO is obtained from GO prepared by using reductive reagents and characterized by its reduced oxygen content and increased hydrophobicity [29]. Compared with GO, rGO has a lower degree of oxidation, lower surface charge, weaker hydrophilicity, and less biological toxicity [30], giving it better biological application prospects, especially in photothermal therapy (PTT). The superior NIR absorption of rGO endows it with high photothermal conversion. Thus, rGO is widely used for PTT in breast cancer [31]. The main limitation of rGO is that most reductive agents used for the reduction of GO are toxic or explosive and difficult to produce on a large scale. Notably, a new environmentally friendly reduction strategy has been designed to solve this problem [32]. Dopamine is one such example, as rGO can be obtained by using dopamine to reduce GO. The stability and dispersivity of rGO can be improved by dopamine surface modification. Yu et al. successfully used dopamine-functionalized rGO composites in PTT for breast cancer [33].

GQDs have the same monoatomic layer structure as pristine graphene and GO sheets but have lateral dimensions of less than 100 nm [34]. Due to their rich surface and edge functions, quantum confinement and edge effects, GQDs, especially solution-treated GQDs, exhibit many novel physical and chemical properties that differ from those of graphene and GO [35]. The two faces and edges of GQD sheets can be used to load drugs, which leads to a loading capacity that is 200% higher than that of other nanodrug carriers [36]. In addition, due to the layered structure of GQDs, atoms, ions and small molecules can be inserted into the graphene layers to improve drug-loading capacity [37]. Notably, compared with graphene and GO, GQDs exhibit stronger photoluminescence properties, which is beneficial both in vivo and in vitro for use as biomarkers in imaging and for other biomedical applications [38]. GQDs have the ability to inhibit multiple drug-resistant genes and show great potential in breast cancer treatment. It has been reported that GQDs can downregulate drug resistance gene expression by interacting with P-glycoprotein (P-gp), the MDR protein MRP1 and the C-rich region of the breast cancer resistance protein gene promoter [39].

### Modifications of GBNs for breast cancer treatment

Reasonable design can allow GBNs to demonstrate amazing performance; such design is mainly concentrated on two aspects: one is design based on the characteristics of breast cancer to obtain better targeting, and the other is design to shape GBNs for better biocompatibility through surface chemistry modification.

### Modifications for targeting effects

#### Active targeting

**Fig. 2:**
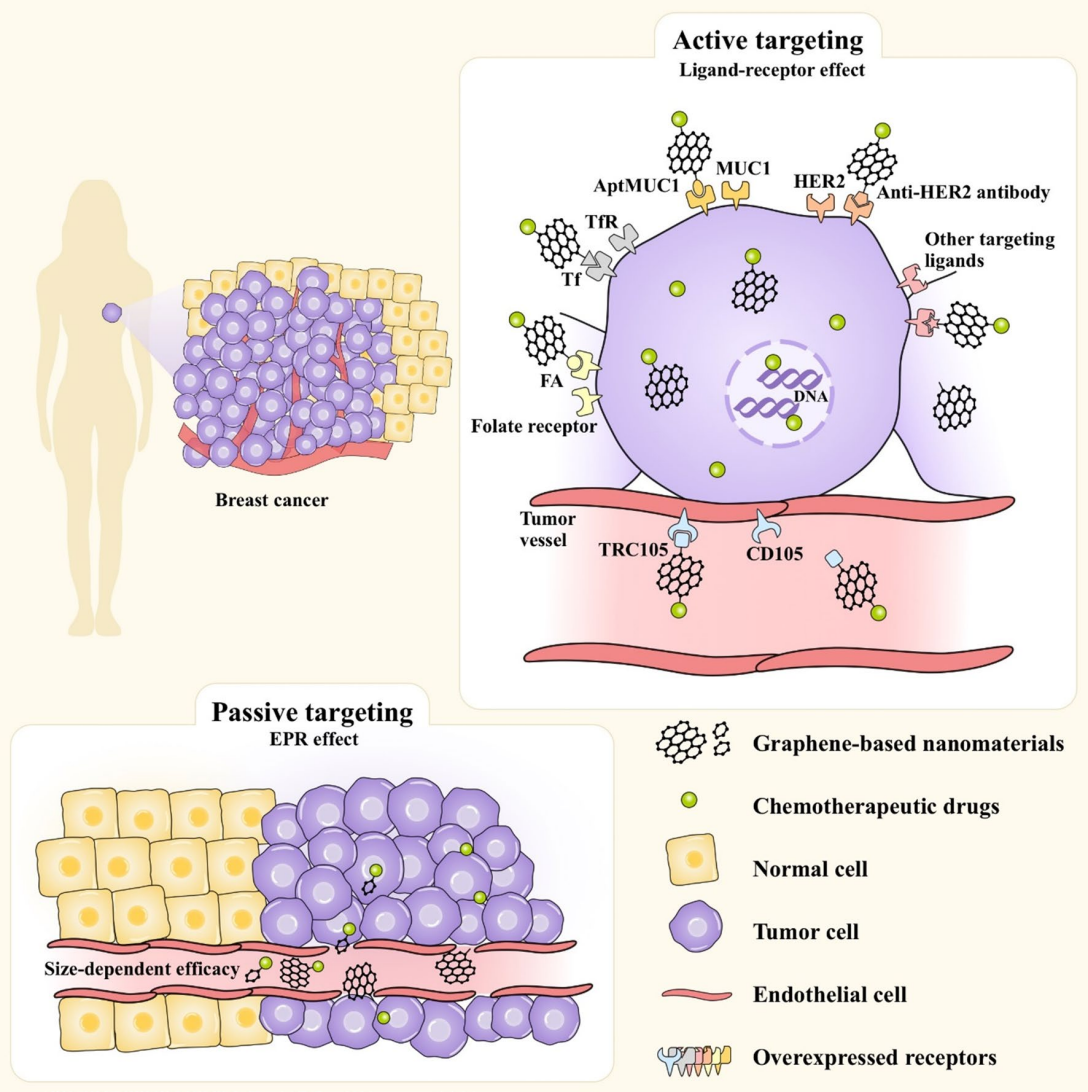
Schematic illustrations of active and passive GBN targeting. Targeting ligands on GBNs are directly attached to a site-specific receptor that is overexpressed on the surface of breast cancer cells or tumor vessels, and the targeting effects of GBNs are then achieved by utilizing receptor-mediated endocytosis and the EPR effect. Abbreviations: FA, folic acid; Tf, transferrin; TfR, Tf receptor; AptMUC1, MUC1-binding aptamer; TRC105, monoclonal antibody that binds CD105; HER2, human epidermal growth factor receptor 2; EPR, enhanced permeability and retention

Active targeting is an effective method to enhance tumor selectivity by coupling tumor-targeting ligands to the GBN surface, which helps to achieve maximum tumor-killing efficacy and significantly reduces nonspecific side effects [40]. Several currently known ligands called targeting agents can target specific molecules on breast cancer cells. Targeting agents work as a coating or are chemically bound to GBNs with loaded drugs. Unlike normal breast cells, breast cancer cells overexpress specific receptors that contribute to the highly selective uptake of drugs into the breast cancer cells (Fig. 2).

### Folic acid (FA)

FA is a B vitamin that is abundant in green plants. FA is transported into cells through folate receptors. Breast cancer cells differentiate and proliferate rapidly, requiring more FA. Thus, folate receptors are overexpressed on the breast cancer cell membrane [41]. FA has the advantages of nontoxicity, low cost, dispersion stability, and target specificity [42]. FA has widely been used to modify GBNs for targeted drug delivery and can enhance the recognition ability and intracellular uptake efficiency of GBNs by breast cancer cells through ligand-mediated targeted effects. Mauro et al. used FA as a targeting agent and employed PEGylated FA-GO in MCF-7 and MDA-MB-231 cells, as it was proven to be selectively recognized by breast cancer cells [43]. In another report, FA and polylactic acid were conjugated to GO. This functionalized GO loaded with doxorubicin (DOX) was administered to MCF-7 cells and significantly inhibited proliferation of the breast tumor cells in a dose-dependent manner [44]. FA and GO were linked though amide bonds formed by condensation of the -NH_2_ groups provided by FA and the -COOH groups provided by GO [45]. Reducing the size of GO and introducing abundant reactive carboxyl groups was shown to efficiently couple more FA and GO [46]. Surprisingly, a study found that FA functionalization enhanced the anticancer effects of GO through toxic responses caused by surface passivation. This may be due to a decrease in the hydrophobic interaction between the breast cancer cell membrane wall and edge of graphene, resulting in destruction of the cancer cell membrane integrity [47]. FA functionalization can improve the biocompatibility of graphene-based drug nanocarriers, making them safer drug-delivery carriers for the treatment of breast cancer [48].

### Transferrin (Tf)

Tf is a hydrophilic single-chain glycoprotein that transports and carries iron that is both absorbed by the digestive tract and released by erythrocyte destruction. Iron transport is achieved through its binding to Tf receptors, which are highly expressed on the membrane of breast cancer cells [49]. Tf is an effective tumor-targeting agent because it is biodegradable, nontoxic, and nonimmunogenic [50]. Tf-functionalized GBNs have been widely applied as drug carriers for breast cancer therapy and proven to increase tumor cell selectivity and significantly increase cytotoxicity to breast cancer cells [51]. Zhu et al. synthesized nanographene oxide (NGO) conjugated with Tf to carry a Pt complex into MCF-7 breast cancer cells. Compared with use of only the free Pt complex, the Pt complex bound to Tf-GO showed greater anticancer effects [52]. Furthermore, Tf protein-surface decoration increased the anticancer activity of therapeutic drugs and reduced the cytotoxicity to normal breast cells due to its specific tumor-targeting ability, assuring its safety and effective usage in vivo.

### The MUC1 ligand

MUC1 is a highly glycosylated protein encoded by the MUC1 gene with a high molecular weight of approximately 250–500 kDa. MUC1 is related to tumorigenicity and cell transformation, and approximately 90% of human breast cancers overexpress MUC1 (> tenfold) [53]. MUC1 is an important breast cancer biomarker that often exhibits a nonpolar distribution in tumor cells and thus has become a target of tumor bioimmunotherapy [54]. MUC1 has been used as a targeting agent in two forms, the MUC1-binding aptamer (AptMUC1) and mucin 1 receptor immunoglobulin G antibody (anti-MUC1 IgG). GO conjugated to anti-MUC1 IgG through a thiol-ene coupling reaction successfully targeted breast cancer and exhibited higher toxicity [55]. Aptamers have several advantages over antibodies, such as their higher affinity for MUC1, nonimmunogenicity, rapid tissue infiltration, low cost and simple synthetic process [56]. The use of AptMUC1 as a targeting agent to functionalize GBNs has garnered increasing research interest. A study utilized AptMUC1 conjugated with GO as the recognition molecule for breast cancer cell internalization and exhibited an excellent targeted apoptosis-induction effect in MDA-MB-231 and MCF-7 cells [57]. GO conjugated with AptMUC1 on its surface showed an ultrahigh density and high flexibility, contributing to the enhanced multivalent binding affinity of AptMUC1 on the breast cancer cell membrane [58]. All of these characteristics have endowed AptMUC1 with its attractive high selectivity for breast cancer treatment.

### Anti-human epidermal growth factor receptor 2 (HER2) antibody

HER2 is a protooncogene related to the growth and invasion of tumor cells [59]. Overall, 20–30% of breast cancers overexpress HER2, with an approximately 100-fold increase in the HER2 protein that leads to uncontrolled cell proliferation. An anti-HER2 antibody (trastuzumab) that acts as a HER2 receptor antagonist can be used to conjugate GBNs and improve their selectivity [60]. Trastuzumab and its conjugates can be absorbed into HER2-positive breast cancer cells via the internalization ability of HER2. Trastuzumab not only specifically guides GBNs to target HER2-positive breast cancer but also suppresses the aggressive behavior and metastatic potential of the tumors [61]. The conjugation of trastuzumab to chemotherapeutics is difficult because trastuzumab is an active macromolecular protein whose active groups should not be occupied when it is combined with other drugs. GBNs can address this limitation by providing a platform on which to conjugate trastuzumab to other chemotherapeutics due to the large surface areas and abundant easily modified groups of GBNs. This is a win–win strategy that not only enables GBNs with a specific targeting ability but also successfully blocks the growth target of breast cancer, making it possible to simultaneously combine targeted therapy and chemotherapy on one platform. Clearly, trastuzumab-functionalized GBNs are attractive carriers since their side effects are minimized due to their significantly increased anticancer efficacy and enhanced targeting specificity. Cornelissen et al. conjugated trastuzumab on 111In-labeled GO for single-photon emission computerized tomography (SPECT) imaging, which showed a synergistic effect between nitrogen-doped GO- and trastuzumab-mediated tumor uptake, resulting in high accumulation in breast cancer cells [62]. However, problems still exist. The sizes of the GBNs decreased after conjugation to trastuzumab because of the crosslinking effect of glutaldehyde. Additionally, as a large number of amino groups are involved in amide bond formation, trastuzumab-rGO also exhibited a less positive zeta potential. These two properties may reduce dispersion stability. In addition, steric hindrance for the interaction of the drug with the GBN surface increased upon trastuzumab binding, which decreased the loading capacity [63]. Thus, trastuzumab-GBNs exhibited loading capacities that were lower than those of unmodified GBNs but still much higher than those of conventional drug carriers. More research focused on improving the drug-loading capacity and stability of trastuzumab-GBNs for the improved application of anti-HER2 antibodies is needed.

### TRC105

CD105 (endoglin) is a homodimeric cell-surface glycoprotein and proliferation-related antigen on the cell membrane [64]. Breast tumor growth requires much neovascularization, so CD105 is highly expressed on breast tumor-associated vascular tissues and the lymphatic endothelium [65]. A high CD105 expression level predicts poor prognosis in breast cancer [66]. One very large advantage of TRC105 (a monoclonal antibody that binds CD105) is that it targets the tumor vasculature instead of tumor cells [67]. The growth, invasion and metastasis of breast cancer are inseparable from the key process of angiogenesis. Due to the lack of drug extravasation requirements, targeting the tumor vasculature is more effective for the delivery of nanomaterials into breast tumors. Thus, functionalized GBNs that target the tumor vasculature are thought to be more attractive than tumor cell-targeting GBNs because they do not require extravasation [68]. Shi et al. conjugated rGO to TRC105 and found that the conjugate specifically targeted 4T1 murine breast tumors in vivo [69]. Another study confirmed that GO can be guided to the tumor neovasculature by targeting CD105, proving that targeting the tumor vasculature is an effective and intelligent method for conjugated GBNs [70].

### Other targeting ligands

The functionalization for GBNs with several novel ligands has been explored; these ligands include octreotide (OCT) [71], follicle-stimulating hormone receptor (FSHR) [72], epidermal growth factor receptor (EGFR) [38], hMnSOD [73], hypericin (HY) [74], fucose [75], and targeting peptides [76]. These ligands emerged into view because of their simple production, high affinity, and special targeting effects. Here, we have chosen to describe some in more detail due to their own unique merits and potential for widespread use with a few related studies. OCT is an analog of somatostatin with the same effects. It can regulate the division, proliferation and apoptosis of breast tumor cells by specifically binding the somatostatin receptor expressed in breast cancer cells. In a study, OCT was used to modify GO as a drug carrier for the specific targeted delivery of DOX to breast cancer cells. Twice as much OCT-functionalized NGO than free NGO accumulated in breast cancer cells, and accumulation of the functionalized NGO compared to free NGO was four or five times higher in the liver and kidney. This good selectivity makes OCT strongly competitive with other targeting ligands [71]. FSHR is a G protein-coupled transmembrane receptor and another highly selective angiogenic marker, such as CD105. The FSHR antibody has the advantages of high affinity, high resistance to degradation, and simple conjugation chemistry. Targeting the tumor vasculature through the FSHR antibody was explored in breast cancer treatment for the first time, and the antibody helped GBNs enter breast cancer cells because targeting tumor blood vessels does not require extravasation. GO conjugated with the FSHR antibody exhibited excellent targeting specificity when compared in FSHR-positive MDA-MB-231 cells and FSHR-negative SKOV-3 cells through flow cytometry and fluorescence microscopy examination [72]. Targeting mitochondria can induce the apoptosis of breast cancer cells by activating the mitochondria-mediated apoptosis pathway, and this process was newly developed as a promising method in breast cancer therapy. GO was reported to increase the production of reactive oxygen species (ROS) and trigger mitochondria-mediated apoptosis. However, permeation into the double membrane of mitochondria is difficult. HY, a product from the extract of *Hypericum perforatum* L., is a novel ligand that targets mitochondria. Han et al. used HY as an effective targeting ligand to functionalize GO loaded with DOX, which was directed to mitochondria and produced a synergistic antitumor effect [74]. However, challenges remain. The utilization of some ligands for cancer detection and treatment is currently at a relatively preliminary stage.

### Passive targeting

Unlike normal tissues, solid tumors such as breast cancer have abundant tumor blood vessels, vascular wall gaps, and poor structural integrity and lack of lymphatic circumfluence, which confers tumor blood vessels with high selective penetration and a high retention time. This phenomenon is called the enhanced permeability and retention (EPR) effect [77]. Passive targeting relies on the EPR effect to achieve material enrichment at the tumor site (Fig. 2). ERP-based passive targeting is the basis for the entrance of GBNs into tumor cells, and the realization of active targeting also includes the EPR effect.

Passive targeting usually utilizes endocytosis or diffusion mechanisms to cross the cell membrane, which requires a prolonged circulation time [78]. The size of GBNs and characteristics of tumor blood vessels are the two main factors that affect circulation time [79]. Enhancing the EPR effect of GBNs can increase active targeting efficacy, which is called the added value of active targeting. Generally, GBNs with a diameter of less than 200 nm can penetrate tumor blood vessels. The size of a nanocarrier plays an important role in the added value of its active targeting. A study showed that small (~ 7 nm) nanocarriers can achieve greater tumor accumulation due to their improved retention. In contrast, no difference in tumor accumulation was observed for larger (~ 14 nm) nanocarriers [80]. Thus, adjusting the size of GBNs and improving their tumor vascular permeability are expected to greatly improve their targeting efficiency. However, the targeting efficacy of EPR-based targeting strategies is limited. Lei et al. invented a new graphene-based targeted nanoprobe that increased the tumor-targeting efficiency up to 50% by changing the permeability of the tumor cell membrane [81]. Thus, the development of new targeting mechanisms to obtain added value to increase the efficiency of passive targeting is feasible.

### Tumor microenvironment (TME) targeting

**Fig. 3:**
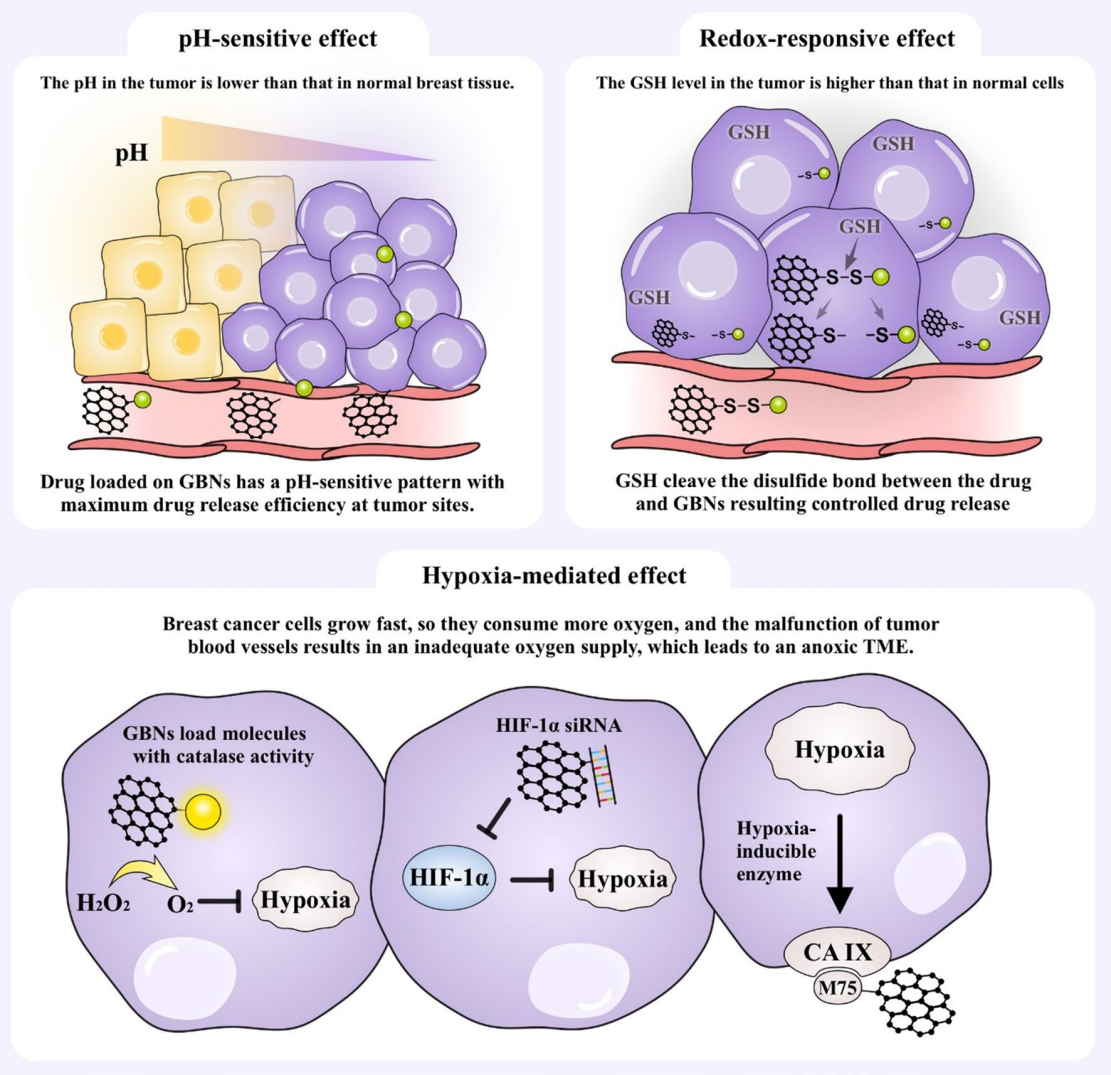
Schematic illustrations of TME targeting. The TME is characterized by hypoxia, slight acidity, a high GSH level and so on. Taking advantage of these characteristics, GBNs can be modified to target the TME for better function. Abbreviations: TME, tumor microenvironment; GSH, glutathione; HIF, hypoxia-inducible factor; -S–S-, disulfide bond; CA IX, carbonic anhydrase; M75, a monoclonal antibody

Hypoxia, slight acidity, and high glutathione (GSH) levels are important characteristics of the TME in breast cancer and play an important role in the development of tumors. Targeting the TME as a therapeutic strategy has gradually become the consensus and a new direction in tumor therapy. In view of this, GBNs can be designed to target the TME (Fig. 3), which would improve perfusion, extravasation and penetration to achieve a greater drug concentration in breast cancer.

### pH-sensitive targeting

Breast cancer cells need an acidic environment to facilitate cell matrix remodeling and accelerate the activity of acid-activating enzymes to meet the demands of tumor growth. The pH in breast tumors is 5.4–7.1, while in normal breast cells, the pH is approximately 7.4 [82]. This difference in pH creates a special tumor microenvironment, which has been used to design pH-sensitive targeted drug-delivery systems. GBNs can be easily conjugated with dynamic covalent linkages, such as acid-liable ester, amide, and ketal/acetal groups, which are pH-sensitive and can be easily cleaved in acidic environments [83]. This pH-responsive release pattern seems to be safe and efficient and can specifically release chemotherapeutic drugs at the tumor site as the result of reduced systemic toxicity and off-target effects [84]. Sericin is a pH-responsive polypeptide that can reverse its charge at approximately pH 5.5 to promote nuclear release. In a study, curcumin was loaded into a sericin-modified GO carrier, and the loaded GO carrier showed excellent pH-dependent release in SKBR3 cells. The pH-sensitive amide linkages were hydrolyzed at pH 5.5, resulting in the rapid release of curcumin [85]. In another study, nanocomposites consisting of gold nanoparticles (AuNPs), FA, dendritic mesoporous silica and rGO to load curcumin were prepared. The maximum release of curcumin occurred at a low pH because both the hydrophobic interactions between curcumin and rGO and electrostatic interactions between curcumin and dendritic mesoporous silica are reduced in acidic environments [86]. L-arginine (L-Arg) is an antioxidant agent with effects against breast cancer cells and therapeutic effects against angiogenesis and apoptosis in tumors. A GO and 5-fluorouracil nanogel crosslinked with L-Arg was found to be degradable and sensitive to pH. At a pH of 5.4, more than 50% of the 5-fluorouracil was released within 6 h, whereas 24% was released at a pH of 7.4. After 24 h, 100% of the drug was released at a pH of 5.4, compared with merely a 32% release at a pH of 7.4 [87]. This pH-responsive drug-release pattern performed well, eliminating breast cancer cells, and showed fewer side effects. Furthermore, this method showed great advantages over traditional methods, but a small amount of chemotherapeutic is still released at a pH of 7.4. A broader spectrum of studies are needed to explore the optimal release mode and to strive for zero release of chemotherapeutic drugs in normal tissues.

### Redox-responsive targeting

During the occurrence and development of breast tumors, the dynamic balance of the cellular redox system undergoes very important changes. The concentration of GSH in breast cancer cells is significantly higher than that in normal cells. Thus, taking advantage of this difference in GSH concentration to trigger redox-responsive graphene-based drug delivery is an attractive strategy. GSH promotes the oxidation of disulfide bonds (-S–S-) [88]. GBNs were linked with drugs via a disulfide bond, and the drug-delivery system was stable in blood vessels; however, when GBNs entered the tumor site, the disulfide bond was cleaved by excess GSH. In a study, GO microcapsules with disulfide bonds on their surface were synthesized and found to display great redox-responsive properties for controlled drug release [89]. The redox environment within tumor cells influences the tumor response to certain chemotherapeutic agents and radiotherapy, so graphene-based redox-responsive targeting strategies could regulate the TME.

### Hypoxia-mediated targeting

Breast cancer cells grow fast, so they consume more oxygen, and the malfunction of tumor blood vessels results in an inadequate oxygen supply, which leads to an anoxic TME. GBNs have been engineered to target hypoxic breast cancer cells. Various strategies have been designed to alleviate tumor hypoxia, including in situ oxygen generation, the inhibition of hypoxia-inducible factor (HIF) expression and the targeting of hypoxia-induced extracellular enzymes [90–92]. The most common approach is to load molecules with catalase activity onto GBNs and to convert excess H_2_O_2_ in breast cancer cells into oxygen. Sahu et al. designed a catalytic GO nanoplatform on which hemin was loaded as a catalase-mimetic nanozyme. This endogenous oxygen-generation system reduced the expression of HIF-1α [18]. Izadi et al. used GO as a carrier to deliver HIF-1α-siRNA into 4T1 breast cancer cells, which suppressed HIF-1α and increased the anticancer effect [93]. Carbonic anhydrase IX (CA IX) is a hypoxia-inducible enzyme that enables tumor cells to adapt to the TME. GO functionalized with the monoclonal antibody M75, which is specific to CA IX, could target cancer cells [92]. Hypoxia has been proven to be related to drug resistance, and relieving breast cancer hypoxia is expected to reverse drug resistance.

### Modifications for better biocompatibility

Concerns about the toxicity of chemically synthesized GBNs in vivo have been raised. Many studies have investigated the biocompatibility and toxicity of GBNs in vivo and in vitro [94]. For example, GO nanosheets were demonstrated to mediate hemolysis [95]. In addition, GBNs can cause cytoskeletal disorders and organelle dysfunction and interact with DNA, mRNA, proteins and other biomacromolecules, inducing apoptosis or necrosis in cells [94]. The main cause of the cytotoxicity of GBNs is their aggregation or coagulation on the cell membrane, which depends on their size, shape and surface geometry [96]. However, GBNs exhibit a wide range of sizes and irregular shapes, which leads different kinds of GBNs to have different toxicological properties [97]. Hence, the application of GBNs in the drug delivery field should focus on modifying their surface chemistry to improve their biocompatibility with cells and biomacromolecules. Strategies to functionalize GBNs, such as coating with polyethylene glycol [98], chitosan [99], or FA [47], have been reported. In addition to the common modifications mentioned above, some new routes have been developed. Mahanta et al. demonstrated that the functionalization of GO with bovine α-lactalbumin (BLA) produced a multifold improvement in its biocompatibility [100]. As another new functional agent, ternary natural deep eutectic solvents (DESs) combined with various functional groups and surface modifications were successfully coupled to GBN nanocarriers and greatly improved the biocompatibility of GBNs. DES-functionalized GBNs can interact with biological organelles in cells and enhance their dispersion stability in aqueous solutions [101]. In summary, strategies such as the use of stabilizers can be explored to avoid the aggregation of GBNs in physiological media and effectively achieve a chemotherapeutic payload.

To design a smart drug-delivery system, ligands should be conjugated with GBNs to simultaneously obtain an active targeting effect that endows the system with stimulus-sensitive controlled release properties. When the drug-delivery system enters the blood circulation and is distributed to all organs of the body, the drug and GBN should be connected by a strong bond that is stable in normal tissues. TME targeting ensures that the drug loaded into the drug-delivery system is rarely released in normal tissue and has minimal toxicity. Then, the targeting ligand guides the drug-delivery system to breast cancer cells or tumor blood vessels, where the TME is different, and the bond rapidly breaks, releasing a large amount of the drug. This leads to a locally high concentration of the anticancer drug. As a result, the designed smart drug-delivery system can effectively kill breast cancer cells with a reduced dose of the chemotherapeutic. Wang et al. modified a quadruple-responsible rGO-based nanocomposite with Rh B and found that the loaded Rh B was released under pH, temperature, NIR light and redox stimulation. In addition, the release efficacy was enhanced by the synergistic effects of multiple stimulus [102]. The multitargeted modification of GBNs will become a trend in the future, and the importance of surface chemical modification of GBNs to improve biocompatibility should not be ignored.

### Imaging and diagnosis

Common and traditional imaging methods for the diagnosis of breast cancer include ultrasound, mammography, magnetic resonance imaging (MRI) and positron emission tomography (PET). However, current imaging methods are not sensitive enough to detect small lesions. The earlier breast cancer can be detected, the greater the possibility of a cure is. Therefore, there is a need to develop new detection methods, such as the use of GBNs in detection applications. Compared with GQDs, bulk graphene itself has worse fluorescence properties. Thus, two strategies have been explored to improve ease of detection. One strategy is to combine bulk graphene with magnetic NPs or fluorescent probes, and the other is to decrease the dimensions of graphene, generating quantum dots.

Bulk graphene modified with magnetic NPs or fluorescent probes can improve the contrast of traditional imaging technologies and enhance the ability to identify and visualize tumor cells that are difficult to detect. For example, magnetic iron oxide nanoparticle (IONP)-modified GO has been employed as a T2 contrast agent for MRI and found to be efficient for cellular MRI [103]. Bulk graphene with fluorescent probes were used in new detection methods, such as cellular imaging, SPECT, PET, and laser desorption/ionization mass spectrometry, and these modified GBNs proved to be good fluorescent diagnostic materials [104]. The specificity and selective sensitivity of fluorescent probes can be provided through conjugation with FA, TRC105, MUC1, or miRNA. In one study, a DNA/GO-sensing platform was used for RT-PCR and fluorescence confocal imaging, and the nanoprobe could recognize different RNA expression levels, which is of great significance for the early diagnosis of breast cancer and monitoring of breast cancer development. This DNA/GO-sensing platform was designed with two different fluorescent dyes labeled with different single-stranded DNA molecules conjugated onto the surface of GO via π–π stacking [105]. Wate et al. used Fe_3_O_4_ NPs and polydendrimers to modify GO as a multicomponent nanosystem loaded with the fluorescent NIR probe cyanine 5.0 for cellular imaging. This nanosystem showed bright and stable fluorescence when internalized by MCF-7 cells [104].

**Fig. 4:**
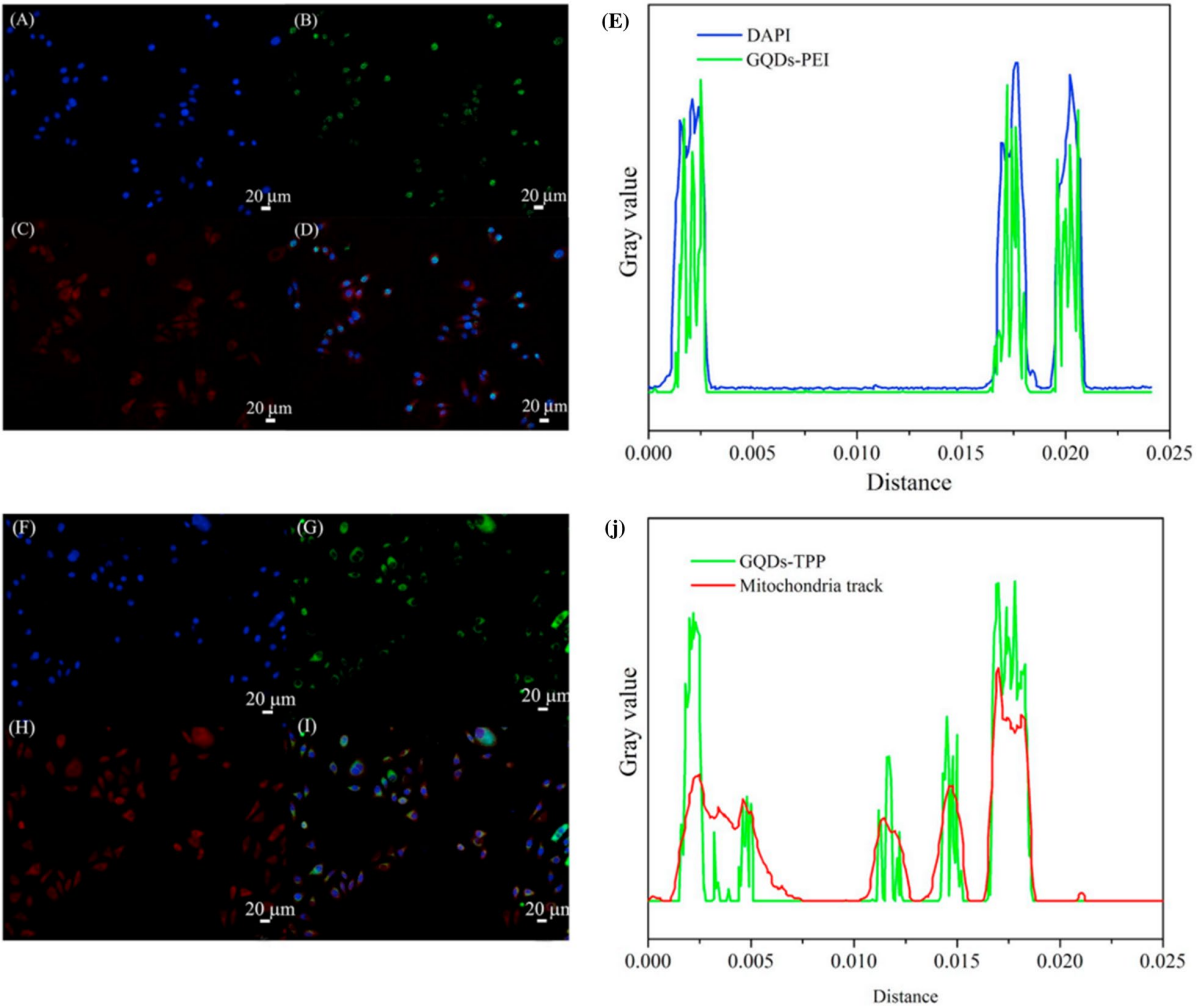
Comparison of the imaging performance of modified GQDs and commercial fluorescent dyes. Fluorescent cell imaging (coculture with cells for 4 h). **A** DAPI, **B** GQDs-PEI, and **C** MitoTracker. **D** Image showing nuclear colocalization between DAPI and GQDs-PEI. **E** Peak positions of DAPI and GQDs-PEI obtained with ImageJ software. (F) DAPI, **G** GQDs-TPP, and **H** MitoTracker. **I** Image of the nuclear colocalization between DAPI and GQDs-TPP. **J** Peak positions of DAPI and GQDs-TPP obtained by ImageJ software [107]. Reprinted from Materials science & engineering. C, 103, Fan Z, Nie Y, Wei Y, Zhao J, Liao X, Zhang J, Facile and large-scale synthesis of graphene quantum dots for selective targeting and imaging of cell nucleus and mitochondria, 109824, 2019, with permission from Elsevier. Abbreviations: GQDs, graphene quantum dots; PEI, polyethyleneimine; TPP, (3-carboxyl) phenyl bromide phosphine

**Fig. 5:**
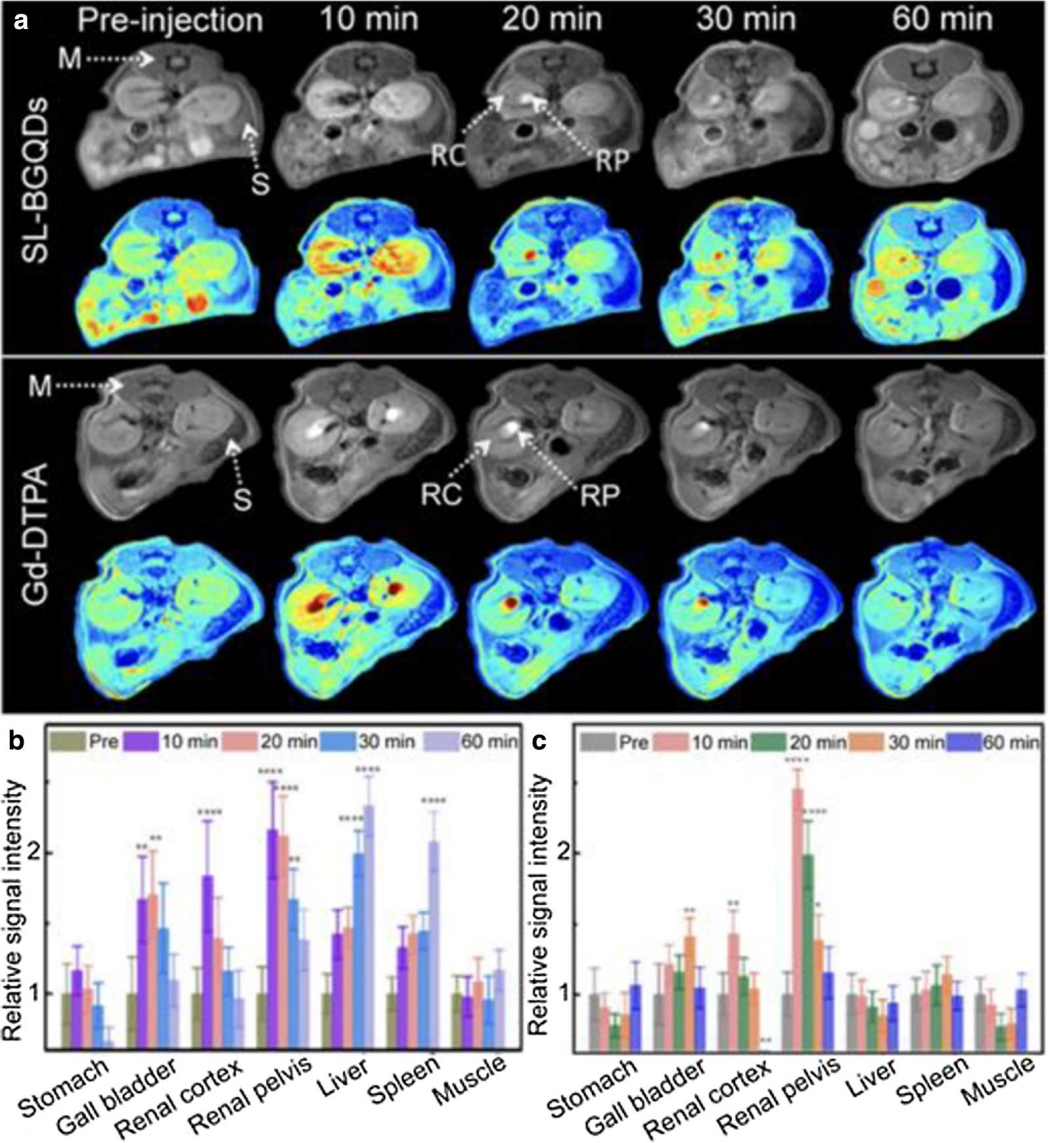
In vivo abdominal MR images of mice intravenously administered SL-BGQDs or Gd-DTPA. T1-weighted MR of the cross-sections of mice that received SL-BGQDs or Gd-DTPA injection acquired using dynamic time-resolved MR imaging at various time points postinjection (**a**) and relative T1-weighted signal intensity (**b** and **c**) [109]. Reproduced by permission from the Royal Society of Chemistry. Abbreviations: SL-BGQDs, boron-doped graphene quantum dots; M, muscle; S, spleen; RC, renal cortex; RP, renal pelvis; Gd-DTPA, Gd diethylene penta-acetic acid

However, the main problems with these fluorescent probes are their light instability, flicker and sensitivity to the environment, which reduce their efficiency. GQDs are an ideal choice to solve these problems due to their characteristics, such as their ability to emit fluorescence at different wavelengths, tunable luminescence, high optical stability and low toxicity [106]. Studies have proven that the imaging performance of GQDs is comparable to that of commercial fluorescent dyes, but GQDs have the advantage of low cost and large-scale preparation. GQDs can selectively target and image the nucleus and mitochondrion. Fan et al. confirmed that GQD-polyethyleneimine (PEI) can selectively stain DNA in the cell nucleus and colocalize with DAPI at the region of the cell nucleus (Fig. 4) [107]. Moreover, the use of GQDs as contrast agents for MRI can improve sensitivity and prolong blood circulation time, especially with doping or functional groups [108]. Gadolinium (Gd)-based chelates are the most commonly used MRI contrast agent but release toxic metal ions in vivo. GQDs, as valid, metal-free MRI contrast agents, could solve this problem and enable higher resolution. A biocompatible GQD-based nanocomposite made from a single-layer graphene quantum dot doped with boron (SL-BGQD) with better contrast than the clinical contrast agent Gd diethylene penta-acetic acid (Gd-DTPA) was, was reported. Compared with Gd-DTPA, SL-BGQD had a significantly higher positive enhancement effect in the imaging of important organs, including the kidney, liver, spleen, and especially blood vessels (Fig. 5). SL-BGQD can cross the blood–brain barrier (BBB), enabling the diagnosis of brain metastases from breast cancer because its imaging time is 50 min longer than that of traditional Gd [109].

Notably, functionalized GBNs have the advantages of low toxicity, high accuracy, higher tumor-to-background ratios, and little extravasation, which confirms the suitability of their application as a valid and suitable approach for breast cancer detection [60]. However, GBNs have rarely been applied in breast cancer imaging or diagnosis. Existing imaging methods, including real-time intraoperative margin imaging in breast-conserving surgery, MRI imaging after neoadjuvant chemotherapy to evaluate tumor changes, and sentinel lymph node imaging biopsy, in which high accuracy in the identification of small lesions is required because such identification is crucial for breast cancer patients’ subsequent treatment options, are not satisfactory. Though GBNs show great prospects to realize accurate breast cancer imaging, more future research to apply GBNs in such imaging is required.

### Various therapeutic strategies involving GBNs in breast cancer treatment

Due to the unique characteristics of GBNs, they can not only lead to breakthroughs in traditional treatment but also show good application prospects in some new treatment methods. Here, we review various therapeutic strategies involving GBNs for breast cancer treatment.

### Delivering chemotherapeutic drugs

Chemotherapeutic drugs have been used as standard treatments for breast cancer but have multiple adverse effects. Neoadjuvant chemotherapy brings hope to many patients with inoperable breast cancer, which increases the rates of breast-conserving therapy and tailors the optimum extent of breast and axillary treatment [110]. The most commonly used chemotherapeutic drugs for breast cancer are members of the anthracycline and taxane families, which include the representative members DOX and paclitaxel (PTX), respectively [111]. However, their shortcomings, including their high cytotoxicity, poor solubility, low bioavailability, nonspecificity, and drug resistance, have limited their therapeutic effects for breast cancer treatment [112]. Some researchers have employed GBNs as drug-delivery systems to promote the utilization and reduce the severe side effects of chemotherapeutic drugs. The applications of GBNs in chemotherapy are summarized in Table 1.

**Table 1:** Applications of GBNs in chemotherapy

References	Type of GBN	Targeting ligands	Modification	Drugs	Properties	Cell line	Animal model	Results
[113]	GO	_	A-Im	DOX	pH-dependent, up to 91% DOX loading	T47D	BALB/c mice bearing 4T1 tumors	Tumors were eliminated 10 days after intravenous injection of GO-A-Im/DOX, and no regrowth was detected
[114]	GO	FA, heparin	PEI, GDC0941	DOX	Dual targeting	4T1	BALB/c nude mice	Both inhibited the primary tumor and suppressed pulmonary metastasis
[115]	GO	_	PEG	DOX	Maximal DOX-loading rate reached, 99.6%	EMT-6	_	Promoted the apoptosis of tumor cells induced by DOX and increased targeting sensitivity and water solubility
[116]	GO	_	_	DOX	pH-sensitive	MCF-7/ADR	_	Reversed drug resistance
[117]	GO	_	F38, T80, MD	EA	pH-dependent	MCF-7	_	Enhanced the cytotoxicity of EA when loaded on functionalized GO
[111]	GO	_	PEG	PTX	High PTX-loading capacity	MCF-7	_	Improved the bioavailability of PTX and showed increased cytotoxicity
[98]	GO	_	PEG	PTX	pH-sensitive	MCF-7	_	Showed increased cytotoxicity
[45]	GO	FA	Methyl acrylate	PTX	_	MD-MB-231	DMBA-induced mammary carcinoma-bearing rats	Alleviated mitochondrial dysfunction in breast cancer
[118]	Graphene	_	_	GA	_	MCF-7	_	Suppressed cellular integrity
[119]	GO	HA	Pluronic	MIT	Acid/NIR laser-triggered and accelerated the release of drugs	MCF-7/ADR cells	_	Overcame multidrug resistance (MDR)
[51]	GO	Transferrin	PAH	DTX	pH-dependent	MCF-7	_	Effectively killed cancer cells
[120]	GQDs	_	_	MTX	_	MCF-7	_	Loaded MTX showed significantly more cytotoxicity than free MTX
[121]	Graphene	_	Chitosan gel	MTX	Thermosensitive	MCF-7		Inhibited the proliferation of MCF-7 cells
[122]	Graphene	_	_	MTX	_	4T1	_	Exhibited a high affinity to ctDNA at wide range of concentrations
[123]	GO	_	β-CD, PVP	CPT	_	MCF-7	_	Efficient CPT-loading, release and breast cancer cell-killing activity
[124]	GO, Porous graphene	_	_	Ginseno-side Rh2	_	MDA-MB	_	Enhanced anticancer activity
[8]	GO	_	_	FU	GO	MCF-7	_	Increased the cytotoxicity of FU
[87]	GO	L-arginine	_	FU	Controlled release at pH 5.4	MCF-7	_	Enhanced the loading and release efficiency of 5-FU
[125]	GO	FA	Sulfonic acid groups	DOX, CPT	Targeted delivery	MCF-7	_	Overcame drug resistance
[40]	rGO	_	PF-127	Curcumin, PTX	High loading capacity	MDA-MB-231	_	Showed high potency against MDA cells
[126]	GO	FA	Chitosan	CPT, DIM	Controlled release	MCF-7	Female albino Wistar rats	Showed synergistic anticancer effects and covered the toxicity of CPT
[127]	GQDs	Herceptin	β-CD, PEG	DOX, Herceptin	Dual pH- and GSH- responsive	SK-BR-3, MDA-MB-231	Xenotransplantation of human breast cancer cells in mice	Significant inhibition of tumor growth
[52]	GO	Transferrin	_	Pt	pH-responsive drug release	MCF-7	_	Exhibited increased anticancer efficacy
[128]	GO	_	_	Pt	_	MCF-7	_	Increased the accumulation of Pt in breast cancer cells

Dox, doxorubicin; EA, ellagic acid; PTX, paclitaxel; CPT, camptothecin; GA, gambogic acid; PAH, poly(allylamine hydrochloride); DTX, docetaxel; MIT, mitoxantrone; MTX, methotrexate; FU, fluorouracil; Pt; platinum; A-Im,1-(10- bromoanthracene-9-yl)-1H-imidazole-4,5-dicarboxylic acid; GDC0941, a phosphatidylinositide 3-kinase/Akt phosphorylation inhibitor; F38, Pluronic F38; T80, Tween 80; MD, maltodextrin; PVP, polyvinylpyrrolidone; β-CD, β-cyclodextrin; PF-127, an amphiphilic polymer

Drugs have been coupled to GBNs simply via π−π stacking and hydrophobic interactions, and these nanosystems exhibited a relatively high loading capacity [98]. Physical adsorption of the drug can maintain its biological activity compared with that upon loading the drug onto the carrier through covalent bonds when a chemical reaction between the drug and carrier is lacking [111]. Some studies have pointed out that GBNs are used as not only carriers but also antitumor drugs because of their intrinsic anticancer activities. Therefore, the combination of drugs and nanostructures cannot be separated, and the whole nanostructure enters the cell as a drug [124]. Moreover, another study reported that only the drug was internalized by breast tumor cells, and the rGO drug-delivery vehicle was left behind, as observed by the presence of rGO outside the cell [111]. In general, GBNs, as chemotherapeutic drug-delivery vehicles, are nontoxic to normal cells and can help to address the limitations of chemotherapy.

### Reducing the toxicity of chemotherapeutic drugs

The use of GBNs as drug-delivery systems can reduce the toxicity of chemotherapeutic drugs. In the clinic, chemotherapeutic drugs require administration at a high dose to maintain an effective concentration, but dose-dependent toxicity commonly limits their use. GBNs can not only deliver anticancer drugs specifically to the tumor site but also increase active targeting to tumor sites, leading to locally high drug concentrations in tumor sites. This contributes to low drug dose requirements and a reduction in drug administration cycles, resulting in the minimization of adverse effects [114, 129]. By binding GBNs, chemotherapeutic drugs have higher antitumor ability than the free drug at the same dose [98]. Thus, the carriers can be used to load a low dose of drug and achieve the same therapeutic effect observed with a high dose of the free drug. The half-maximal inhibitory concentration (IC50) is commonly used as a measure of drug effectiveness. The low IC50 values of graphene-based drug-delivery systems result in enhanced cytotoxicity toward breast tumor cells in vitro [46]. Ashjaran et al. loaded fluorouracil into GO/NHs and found that this system could decrease the IC50 of fluorouracil in MCF-7 cells, showing that the GBN drug carrier reduced the treatment dose of fluorouracil with no impact on efficiency [8]. The drug-delivery system was also beneficial in limiting the uncontrolled release of chemotherapeutic drugs in the biological system and helped to decrease possible side effects [128]. Saeednia et al. designed a thermosensitive hybrid hydrogel consisting of chitosan and graphene, which was used to control the delivery of methotrexate. The addition of graphene to the thermosensitive chitosan hybrid hydrogel decreased the cumulative release of methotrexate, which made the release slower and more controllable [121]. Additionally, pH-responsive graphene-based drug carriers were designed to achieve controlled drug release. A study using L-Arg conjugated to GO to deliver fluorouracil into breast cancer cells proved the controlled release of fluorouracil at pH 5.4 [87]. A scientific drug-controlled release model could enhance the anticancer effects of chemotherapeutic drugs, leading to a reduction in dose-dependent toxicity.

### Increasing the solubility and bioavailability of chemotherapeutic drugs

The use of GBNs as a drug-delivery system can increase both the solubility and bioavailability of chemotherapeutic drugs. Most anticancer drugs are either insoluble or only slightly soluble in water because of their hydrophobicity. However, they can be combined with highly polarized groups to become hydrophilic [123]. Therefore, the introduction of GBNs can improve the water solubility of these drugs, enhance their ability to cross the target cell membrane to increase their uptake into breast cancer cells, improve their stability and prolong their shelf life [130]. For improved pharmaceutical applications, GBNs can covalently bind hydrophilic and biocompatible excipients to improve the solubility of these GBN-loaded drugs. Yan et al. synthesized a GO-PEG-DOX complex and proved that PEG functionalization can improve the water solubility and increase the tumor-targeting sensitivity of DOX, resulting in a stronger DOX-mediated breast tumor-killing effect [115]. The observed significant improvement in the solubility of chemotherapeutic drugs is believed to be mainly due to the presence of GBNs and their excipients. Furthermore, an increase in drug solubility will increase bioavailability. The bioavailability of GBN drug carriers is enhanced due to their high drug-loading capacity as a result of their large surface area. A large number of drugs can also be adsorbed onto GBNs because of the hydrogen bonding interactions between GBNs and drugs [131]. The EPR effect induced by GBNs can increase drug accumulation in breast tumors, and GBNs also perform well in tumor vasculature extravasation [132]. In summary, GBNs can be used to design a drug-delivery system with a long blood circulation time, highly selective targeting effect and controlled drug-release mode to improve the local tumor drug concentration and bioavailability.

### Increasing the specificity of chemotherapeutic drugs

The inability to specifically deliver drugs to tumor sites is the main problem of chemotherapeutic breast cancer treatment and leads to unavoidable damage to normal cells due to the lack of specificity. GBNs can increase the specificity of chemotherapeutic drugs through active targeting and passive targeting [133]. Active-targeting therapy involves the conjugation of targeting agents onto the surface of GBNs. These targeting agents can be specifically recognized by breast cancer cells to increase the specificity of the drugs loaded onto the GBNs [26]. Targeting agents, such as FA, Tf and fucose, are administered through ligand-mediated drug delivery to increase the intracellular uptake of the chemotherapeutics by breast cancer cells. The EPR effect endows GBNs with passive targeting abilities [134]. Passive targeting can utilize endocytosis or diffusion mechanisms to cross the cell membrane due to the vast number of pores and poor lymphatic drainage in breast tumor sites [135]. The sizes of GBNs and characteristics of tumor blood vessels are the two main factors that affect passive targeting [136]. Drugs loaded onto GBNs can show good tumor vasculature extravasation, which promotes the selective distribution of drugs into tumor tissues, resulting in an increase in the efficacy and a decrease in the side effects of the system [92]. A study used PEG-functionalized graphene sheets, which showed a highly efficient passive breast cancer-targeting effect and low retention in reticuloendothelial systems. These properties are attractive for the design of drug-delivery systems [48].

### Reversing drug resistance

MDR is the bottleneck of breast cancer chemotherapy. Conjugation with GBNs has been studied as a remarkable and novel strategy to solve this problem. Several molecular mechanisms of MDR have been revealed, including decreased drug uptake, the activation of DNA repair, the escape of drug-induced apoptosis, enhanced detoxification activities and enhanced expression of the P-gp transporter [78]. In detail, these enhanced detoxification activities acquired by drug-resistant cells can transform chemotherapeutics into innocuous derivatives, and their anticancer effects are diminished as a result [79]. GBNs are helpful in overcoming the enhanced detoxification activities of drug-resistant cells by increasing cellular uptake, resulting in enhanced drug absorption. A study proved that GO-loaded adriamycin could reverse the drug resistance of MCF-7/ADR cells that are resistant to adriamycin by increasing its cellular uptake while also showing good biosafety [116]. In another MDR mechanism, the enhanced multidrug resistance gene in drug-resistant cells promotes encoding and expression of the P-gp transporter, which pumps drugs out of breast cancer cells and fails to kill them [137]. The strategy of designing a drug carrier that can codeliver chemotherapeutics and P-gp inhibitors is a valid method, but the carrier needs to be nontoxic and must have the ability to increase therapeutic effects [138]. GBNs can silence multiplex genes to inhibit P-gp expression and reverse MDR. Li et al. used two molecular beacons to modify GO and reverse MDR. These molecular beacons hybridized with target sequences could decrease the efflux of DOX by decreasing expression of the P-gp transporter [139]. Another strategy is to avoid P-gp export effects to reverse MDR in breast cancer cells [119]. GBNs are taken into breast cancer cells in both endocytosis-dependent and endocytosis-independent manners. GBNs can enter cells by endocytosis and then release drugs into the cytoplasm at low pH levels in endosomes or lysosomes, which effectively avoids the export effects of surface-expressed P-gp in MCF-7/ADR cells [139]. Adriamycin localizes to the nuclei of breast cancer cells by escaping the recognition and export effects of P-gp when GO is used as a carrier, resulting in the reversal of drug resistance [140]. These results suggest that the utilization of GBNs as chemotherapeutic carriers is attractive for MDR breast cancer treatment.

### Codelivering drugs

Functional GBNs as nanocarriers of mixed anticancer drugs are an efficient strategy to fight breast cancer. Codelivery, a widely adopted clinical practice, is the combined use of two or more chemotherapeutics [141]. Codelivery has the clinical potential to produce a synergistic effect from the different mechanisms of action of multiple drugs against breast cancer. Compared with delivery of a single drug, codelivery showed improved therapeutic efficacy to inhibit breast cancer cell proliferation and a reduced required dose of each drug. Moreover, GBNs can solve the problem of the easy excretion of drugs due to their low molecular weight [125]. Ko et al. designed a multifunctional nanosystem in which Herceptin and DOX dual delivery was achieved by using PEG-functionalized GQDs for the treatment of HER2-positive cancer. This nanosystem displayed a synergistically enhanced antitumor effect both in vivo and in vitro [127]. The premise of codelivery is that the bioavailability and activity of each drug are not affected [40]. Due to the different drug-release sequences and release effects, the drug-delivery system should have a controlled release mode that is different from that of free drug. For example, the advanced release of curcumin can achieve a chemosensitization effect in breast cancer cells, thereby improving the therapeutic effects of PTX [142]. Deb et al. used chitosan and FA to functionalize GO to simultaneously deliver camptothecin and 3,3'-diindolylmethane into MCF-7 cells [126]. The results showed that codelivery could help to overcome the toxicological effects of free camptothecin and enhance its anticancer activity. The use of GBNs to control the loading and targeted release of mixed anticancer drugs has broad application prospects in biomedicine.

### Gene-based therapy

Gene therapy is based on the introduction of nucleic acids into targeted cells through gene knockout or expression [143]. Gene therapy solves the root of the problem of gene abnormalities, breaks through traditional treatment methods and can be developed into precision medicine. A carrier to transport and protect nucleic acids from degradation during transportation due to the properties of genes, including their negative charge, unstable structure and poor cellular uptake, is needed [144]. Viral and nonviral vector systems are two strategies for gene delivery. The efficiency of viral vector gene transfer is high, but there are some deficiencies, such as high cost, the host immune response and insecurity [145, 146]. The preparation of nonviral vectors is simple, and the risk of an immune response is low. However, due to the influence of many extracellular and intracellular barriers, its transfection efficiency is always low [147]. Due to the limited ability of siRNA to cross the cell membrane, several kinds of nanodelivery systems have been developed as nonviral vectors to promote siRNA entry into the cytoplasm of breast cancer cells [148]. The ideal vector not only concentrates genes effectively but also protects genes from degradation by nucleases [149]. GBNs might be suitable candidate vectors. For example, GO protects siRNA from enzyme digestion and shows satisfactory transfection efficiency. It also has the advantages of low cost and easy mass production [150]. The applications of GBNs in gene-based therapy are summarized in Table 2.

**Table 2:** Applications of GBNs in gene-based therapy

References	Type of GBN	Targeting ligands	Modification	Gene	Properties	Cell line	Results
[151]	GO	_	PEI	siRNA	_	MDA-MB-231	Suppressed the gene expression and metastatic potential of breast cancer cells
[152]	GO	_	CPPs, PL-PEG	siRNA	_	MCF-7	Showed significant cell death
[153]	GO	P-L-Arg	Au	miRNA-101	Gene release was improved by NIR thermal therapy	MCF-7, MDA	Reduced the viability of MCF-7 cell lines
[154]	GO	P-L-Arg	PEG	miR-101	Higher miRNA payload, selective transfection	MCF-7, MDA-MB-231	Autophagy was downregulated, and the apoptosis cascade was activated
[155]	GO	_	Hap, ganciclovir	HSV-TK	Tumor-specific promoters	MCF-7, MDA-MB-231, MCF-10A	Induced the apoptosis of breast cancer cells and inhibited their growth
[156]	GO	_	PEG-diamine, R8	siRNA and pDNA	Superior internalization efficacy of 85%	MCF-7, MDA-MB-231	Protected siRNA and pDNA against enzyme degradation
[139]	GO	_	_	MDR1 beacon, ETS1 beacon	_	MCF-7/Adr	Reversed MDR
[150]	GO	_	PAMAM, PEG	siRNA	pH-triggered release of siRNA	MDA-MB-231	Effectively silenced genes with high transfection efficiency

PEI, polyethylenimine; siRNA, small interfering RNA; CPPs, cell penetrating peptide; PL-PEG, a phospholipid-based amphiphilic polymer; Au, gold; P-L-Arg, poly-L-arginine; miR-101, microRNA-101; PEG, polyethylene glycol; HSV-TK, herpes simplex virus thymidine kinase gene; Hap, hydroxyapatite; PEG-diamine, aminated-polyethylene glycol; R8, octaarginine; MDR1, multidrug resistance 1; ETS1, erythroblastosis virus E26 oncogene homolog 1; PAMAM, polyamidoamine; GPD, hybrid vector consisting of GO, PAMAM and PEG

Many regulatory factors, such as siRNA [152], pDNA [156], microRNA-101 (miR-101) [153]and the herpes simplex virus thymidine kinase gene (HSV-TK) [139] have been used in graphene-based gene therapy for breast cancer treatment. Huang et al. produced PEI-functionalized GO to transfect siRNA against invasive breast cancer cells. The results indicate that PEI-functionalized GO is effective in siRNA delivery and may be helpful for targeted gene therapy to inhibit breast tumor metastasis [151]. The prepared nanoplatform GO-PEG-(P-L-Arg) was reported to deliver miR-101, showing a high miR-101 payload and internalization in breast cancer cells. In this study, the nanoplatform showed highly selective transfection due to the targeting effects of P-L-Arg, which significantly reduced the side effects [154]. The use of GBNs to transfer transcriptional regulatory elements is an effective method of gene therapy. For example, estrogen response elements and hypoxia-responsive elements have been used to direct the expressed genes to breast tumors [157, 158]. Cheang et al. used GO to deliver estrogen response elements and hypoxia-responsive elements to increase the expression of HSV-TK in breast cancer cells, inhibiting proliferation and inducing apoptosis. This contributed to noteworthy antitumor effects [139].

### Phototherapy

Phototherapy is a noninvasive treatment that does not produce scars and heals quickly. PTT and PDT are representative phototherapies with great potential to overcome the limitations of conventional chemotherapy in cancer treatment [159]. GBNs with light-induced properties have been explored for the phototherapy of breast cancer cells. The applications of GBNs in phototherapy are summarized in Table 3.

**Table 3:** Application of GBNs in phototherapy

References	Type of GBN	Targeting ligands	Modification	Treatment	Properties	Cell line	Animal model	Results
[160]	GO	_	Gold nanostars	PTT	Low laser power	SK-BR-3	_	Improved the photothermal effect
[161]	GO	MUC1	Apt-AuNP	PTT	Induced a transient increase in HSP70 expression	MCF-7	_	Inhibited the growth of MCF-7 cells
[162]	GO	_	TPGS	PTT	Produced a decrease in the levels of BcL-2 and Survivin	MCF-7	_	Reduced the viability of breast cancer cells with no obvious effect on normal cells
[31]	rGO	Arg	_	PTT	Excellent NIR absorption	MDA-MB-231	_	Increased cellular uptake and enhanced the photothermal effect
[33]	rGO	_	pDA, AAP10	PTT	Bystander effect	MCF-7	Mouse 4T1 breast tumor model	Showed excellent anticancer activity
[163]	rGO	_	Ag-ZnO	PTT	Low-level laser therapy	MCF-7	_	Showed a considerable killing effect at a low concentration and with a low-level laser
[164]	rGO	_	PEG	PTT	Low toxicity	4T1	4T1 tumor-bearing BALB/c	Increased tumor accumulation and enhanced the PTT effect
[165]	rGO	_	Tamoxifen citrate	_	_	MCF-7	Female BALB/c nude mice MCF-7 cell lines	Thermally induced necrosis of breast cancer cells
[166]	rGO	_	Fe_3_O_4_ NPs, PEG	PTT, Immunotherapy	MRI-guided	4T1	4T1 orthotopic mouse	Laser irradiation effectively ablated primary tumor and induced antitumor immunity
[167]	GO	_	CuSQDs	PTT	Downregulated Bcl-2 expression	MCF-7	_	Significantly enhanced cytotoxicity against MCF-7 cell lines
[168]	GO	_	Ag	PDT	_	MCF-7	_	Produced significant toxic effects in MCF-7 cells
[169]	GO	_	HPPH, PEG	PDT, immunotherapy	_	_	4T1 murine breast cancer mode	Prevented distant metastasis
[170]	GO	_	MB	PDT	_	MDA-MB-231	_	Showed enhanced PDT efficiency
[171]	GQDs		hMSN, PEG	Chemotherapy, PDT	pH-dependent	4T1	Female BALB/c mice	Prolonged the retention time in tumor
[172]	GQDs	_	MB	PDT	_	MCF-7	_	Showed no change in cytotoxicity or ROS production

Apt-AuNPs, aptamer-gold nanoparticles; HSP, heat shock protein; TPGS, D-α-tocopheryl polyethylene glycol 1000 succinate; pDA, polydopamine; AAP10, antiarrhythmic peptide 10; CuSQDs, copper sulfide quantum dots; Ag, silver; HPPH, a photosensitizer; MB, methylene blue; hMSNs, hollow mesoporous silica nanoparticles

### PTT

PTT is a cancer treatment technique in which breast tumors are exposed to a laser and the received light is converted into heat to ablate tumors. Increasing the temperature of breast cancer cells to over 40 °C inactivates many intracellular structural and enzymatic proteins and leads to irreversible tumor destruction [173, 174]. Another advantage of PTT is that this damage is limited to specific areas, so healthy tissue can be preserved. The application of PTT in clinical breast cancer treatments has been confronted with limitations, such as the low penetration depth of NIR irradiation into tissues and low photo-to-heat conversion efficiency. This leads to incomplete tumor ablation and therefore a high risk of recurrence [175]. To address these limitations, high absorption by photosensitizers in the NIR region is required, and these photosensitizers need to be selectively absorbed by breast cancer cells rather than normal cells to lead to the precise heating of tumor cells [176]. PTT employs nanotransducers as photosensitizers, which can efficiently convert photon energy to thermal energy. The excellent NIR optical absorption behavior and enhanced surface activity of GBNs endow them with the potential to be used as photothermal therapeutic agents in breast cancer treatment [177]. The introduction of targeting agents on GBNs was used to obtain specific targeting capabilities and improve the ablation efficacy of PTT [31]. In addition, compared to the individual components alone, graphene-based multifunctional hybrid nanomaterials such as GO and gold nanostar hybrids have greater photothermal effects [160]. It has been reported that the degree and duration of HSP70 protein expression are related to the effects of PTT against breast cancer. Apt-AuNP-hybridized GO as a photosensitizer combined with an HSP70 inhibitor produced a synergistic PTT therapeutic effect, which was proven to be more efficient than the effect of GBNs alone [161]. To obtain deep penetration, high-power NIR irradiation is often utilized. However, high-power irradiation can cause damage to adjacent normal tissues, especially during long-term irradiation. Nanocomposites composed of GBNs showed a rational killing effect in breast cancer cells even with a low-level laser. Ag/ZnO- and Nd/ZnO-functionalized rGO exposed to a 630-nm laser rather than the usual 810-nm laser were proven to be an effective drug-free approach to kill MCF-7 cells [163]. Breast tumors can shield some tumor cells from NIR radiation due to their three-dimensional structure. GBNs can increase the destruction rate of breast tumor cells by enhancing the bystander effect, which transfers the toxic signal of the individual damaged cells to adjacent cells. Yu et al. successfully developed polydopamine-functionalized rGO to enhance the bystander effect to solve the problem of partial tumor penetration [33]. A study used tamoxifen to modify nanosized rGO, which combined hormonal therapy with PTT and showed improved stability and low cytotoxicity. PTT takes advantage of the ability of hormonal therapy to target hormone receptors, which enables precise tumor ablation [165].

### PDT

PDT is a promising local breast cancer treatment strategy that uses a photosensitizer to absorb NIR light and convert excited energy to produce ROS. The production of ROS leads to tumor destruction [178]. Most of the photosensitizers used in PDT have poor water solubility and exhibit a limited ability to permeate cell membranes [179, 180]. Therefore, great attention has been given to the preparation of GBNs, which can optimize the properties of photosensitizers and increase apoptosis and death through PDT. GBN systems have the advantages of improving the transfer of insoluble photoactive agents and promoting the transmembrane transfer of photosensitizers [181]. Due to these attractive advantages of the intrinsic optical properties of GBNs, one strategy is to utilize GBNs themselves as photosensitizers. There have been reports on the phototoxicity of GBNs in breast cancer cells. In an experimental study, GO-silver NP nanocomposites showed cytotoxicity and photodynamic effects toward MCF cells [168]. GQDs exhibit the largest emission in NIR light. Due to their excellent photoluminescence properties and efficiency in generating singlet oxygen, GQDs also show great potential in breast cancer PDT. Future investigations in breast cancer treatment are encouraged due to the similarly considerable photodynamic effects of GQDs [182]. Yang et al. incorporated GQDs into the cavity of hollow mesoporous silica NPs to form a hybrid nanoplatform. These nanocomposites solved the problem of rapid clearance of GQDs through the renal system while exhibiting similar singlet oxygen-generation efficiency [171]. Another strategy is to load photosensitizers onto GBNs via electrostatic and π–π stacking interactions or hydrophobic cooperative interactions [170]. GQDs loaded with a photosensitizer could produce more singlet oxygen than that produced by any of the components alone and enhanced toxicity in breast cancer cells [183]. The synthesis composite ratio has a great influence on the singlet oxygen production output. Methylene blue is a common photosensitizer used in PDT. A study proved that the free form of methylene blue increases within decreasing GO concentration, which leads to decreased PDT effectiveness. Therefore, it is necessary to modulate the ratio of photosensitizer and GO to find a suitable composition [170, 172]. Tumor integrin αvβ6-targeting peptide-functionalized GO loaded with a photosensitizer (HPPH) was applied in PDT of subcutaneous and lung metastatic mouse models. The necrotic breast cancer cells induced by PDT could increase the infiltration of CD8^+^ T lymphocytes, which further activated dendritic cells to inhibit the growth of primary breast tumors and lung metastasis [169]. The use of GBNs as photosensitizers or as carriers to deliver photosensitizers to breast tumor cells can improve the effectiveness of PDT, making it possible to combine PDT with other treatments. Compared with that in combined PDT/PTT treatment groups, undesirable necrosis was found to be increased in groups administered PDT alone or PTT alone, which showed that combined PDT/PTT is a cleaner treatment.

### Combined phototherapeutic strategy

Absorption in the strong NIR visible light range and their high surface area make GBNs suitable for loading with both hydrophilic and hydrophobic molecules to act as photosensitizers. Together, these properties may allow for the possibility of combining PDT and PTT in a single GBN device [184, 185]. This combined phototherapy strategy performed surprisingly well in preventing breast cancer metastasis by promoting breast tumor ablation and stopping breast tumor progression. In addition, metastasis seemed to be hampered after combined phototherapy treatment. The use of NanoGO-methylene blue induced more toxicity in breast cancer cells than in normal breast cells by combined PDT/PTT. Photoacoustic (PA) imaging uses an ultrasound signal generated by light excitation to image the structure and function of biological tissue and is especially suitable for the early detection of cancer and treatment monitoring. The combined use of PA imaging with PTT is another effective strategy. Hu et al. applied polydopamine-functionalized rGO to load indocyanine green for PA imaging-guided PTT in 4T1 breast cancer cell subcutaneous and orthotopic mouse models. The obtained composites exhibited a stronger PTT effect and higher PA contrast than pure rGO or polydopamine-rGO [186]. These results demonstrate that the combined phototherapy strategy is a promising strategy for breast cancer treatment.

### MTT

MTT is a new clinical method for the treatment of breast cancers. Breast tumors are placed under an external alternating magnetic field and killed though the thermal effect induced by magnetic NPs. The applications of GBNs in MTT are summarized in Table 4.

**Table 4:** Application of GBNs in magnetothermal therapy

References	Type of GBN	Targeting ligands	Modification	Drugs	Properties	Cell line	Animal model	Results
[187]	GO	_	PEI, IONPs	DOX	Intratumorally injected	MCF-7	BALB/c mice	Led to more apoptosis and demonstrated higher antitumor efficacy combined with AMF
[188]	rGO	_	SPIONs, PEG	_	_	MCF-7	_	Enhanced the magnetothermal ablation efficacy
[189]	GO	HA	_	DOX, PTX	Targeted delivery	MDA-MB-231, BT-474	_	MTT was used to enhance the efficacy of chemotherapeutics in killing tumor cells
[190]	GO	_	FVIOs	_	Combined magnetothermal effect and ROS-related immunologic effect	4T1	A mouse 4T1 cell subcutaneous breast tumor model	Eliminated the tumor at a low dose and short time under AMF exposure

PEI, polyethylenimine; IONPs, iron oxide nanoparticles; FVIOs, ferrimagnetic vortex-domain iron oxide nanorings; AMF, alternating magnetic field

For the magnetothermal ablation of breast tumors, the temperature usually increases to 37 ~ 45 °C or higher than 45 °C [191]. Graphene itself is not magnetic, so it needs to bind magnetic particles. One example of MTT is superparamagnetic IONPs anchored to rGO. rGO nanosheets have thermal conductivity, which is beneficial for distributing heat evenly to promote tumor ablation [192]. Compared to PDT and PTT modalities, MTT has the advantages of deep tissue penetration and magnetic selectivity to kill breast cancer cells without injuring the surrounding healthy breast tissues [193]. By coupling targeted ligands such as hyaluronic acid to GBNs, the selectivity of MTT can be further improved, increasing the attraction of MTT [189]. Furthermore, PEI- or PEG-functionalized rGO showed improved compatibility and magnetothermal properties [187, 188]. Alhasan et al. synthesized PEGylated rGO to bind superparamagnetic IONPs, which were used under an external magnetic field for MCF-7 breast tumor cell ablation. In the absence of anticancer drugs, such a design significantly enhanced the magnetic thermal ablation of breast cancer [188]. The disadvantage of conventional MTT is that it depends only on the heating effects of the magnetic NPs. Liu et al. introduced magnetothermodynamic (MTD) therapy by combining MTT and immune effects related to ROS to overcome the limited therapeutic effects of MTT. In a 4T1 cell subcutaneous tumor model, a mixture of ferrimagnetic vortex-domain iron oxide nanorings and GO was used as an effective MTD agent, and the symbiotic combination of heating effects and ROS-related immune effects was used to effectively eliminate the tumor at a physiologically tolerable temperature [190]. MTT is expected to be combined with other therapies, which can provide new ideas for breast cancer treatment.

### As a platform for different therapies

**Fig. 6:**
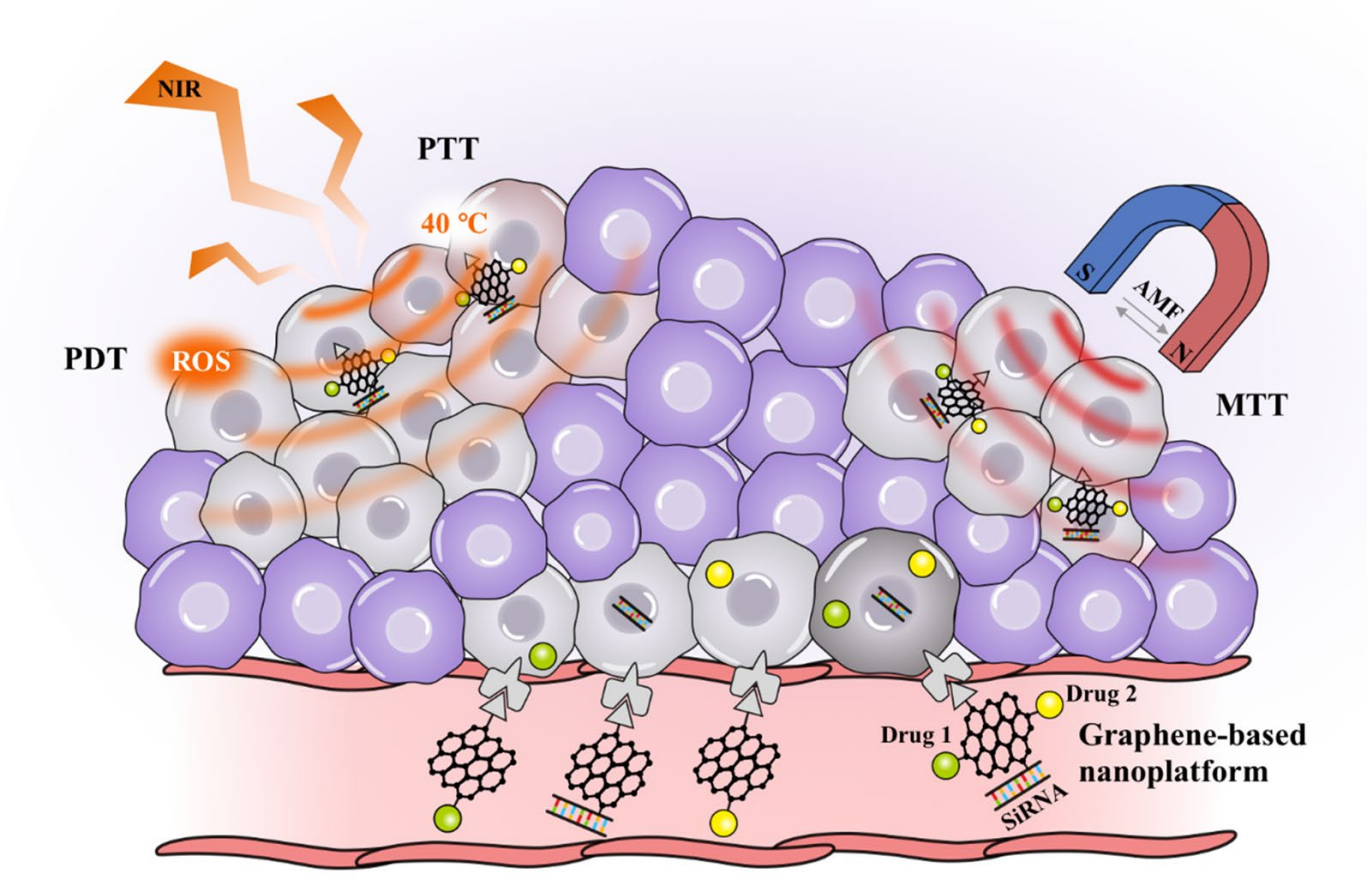
Schematic illustrations of the graphene-based nanoplatform**.** The graphene-based nanoplatform incorporates various therapeutic strategies into one multifunctional platform to achieve optimal therapeutic effects. For example, a graphene-based platform is not only capable of simultaneously loading multiple drugs and genes but can also be used in phototherapy and MTT at the same time. Abbreviations: NIR, near-infrared; PTT, photothermal therapy; PDT, photodynamic therapy; ROS, reactive oxygen species; MTT, magnetothermal therapy; AMF, alternating magnetic field

Although GBNs have great prospects in the treatment of breast cancer, nanodrugs that work through only one therapeutic mechanism may fail and lead to drug resistance. Therefore, a multieffective therapy aimed at killing breast cancer cells in different ways is expected. Combination therapy that uses a graphene-based platform is regarded as a valid anticancer therapeutic strategy to overcome the limitations of treatment with a single therapeutic [194]. Designing such a platform requires finding a biocompatible material with a large surface area that is able to simultaneously load different drugs and genes. This platform should also have light-induced properties. Therefore, GBNs may be suitable. One of their advantages is that graphene-based platforms can exhibit all of the desirable features of each treatment mechanism, and these different treatment methods are concentrated into a single, easy-to-prepare intelligent drug-delivery system [195, 196] (Fig. 6). Compared with single therapies, this platform displays an improved therapeutic effect, the reduced possibility of drug resistance and fewer side effects [197].

Graphene-based platforms with two or more therapeutic effects, including synergistic multichemotherapy [198], chemothermal therapy [199, 200], chemogene therapy [201, 202], chemo-PDT [203] and thermal gene therapy [153], have been studied for breast cancer treatment and can induce the apoptosis of breast cancer cells and inhibit breast tumor tissue growth. Among these studies, graphene-based chemo-phototherapy for breast cancer has been the hottest research topic and has been widely studied. It can not only improve the loading capacity and pharmacokinetics of chemotherapeutics but also release the loaded drugs at a controlled rate through the photothermal trigger process. In addition, GO absorbs NIR radiation and converts it into heat, thus inducing sensitization to the action of chemotherapeutics [204]. Gadeva et al. used quercetin to reduce FA-functionalized GO to achieve chemo-PTT in triple-negative breast cancer. The platform performed well in MDA-MB-231 cells and showed the advantages of high cellular uptake and strong cytotoxic effects, especially under NIR (808 nm) laser irradiation [42]. A multifunctional platform for synergistic breast cancer therapy with controlled DOX release, MTT, and PTT consisted of GQDs and magnetic mesoporous silica NPs showed a significant synergistic effect to kill breast cancer cells with improved efficacy [200]. Treating MDR breast tumor cells with both chemotherapy and gene therapy is an effective approach. Gu et al. used GO to codeliver DOX and MMP-9 shRNA for breast cancer treatment, which had a more significant tumor-killing effect than monotherapy [140]. However, no related study has investigated the suitable ratio of chemotherapeutic drugs to gene drugs, and whether these drugs would exhibit mutual interference is unknown. Future work is needed to explore this question.

The superior antitumor performance of the graphene-based platform implies its good potential in metastatic breast cancer treatment. Wang et al. synthesized a platform including rGO conjugated with polydopamine and gold nanostars to load DOX, which, after exposure to laser irradiation, could synergistically inhibit the growth of primary tumors and reduce the number of pulmonary metastatic nodules in the lungs of a metastatic 4T1 cell breast tumor model. Furthermore, unlike the free DOX-treated mice, no metastasis was observed in the livers of the mice with this platform treated with a laser, showing the ability of the platform to suppress distant metastasis [205]. A platform composed of a tumor integrin αvβ6-targeting peptide, GO and a photosensitizer was explored to trigger host immunity with tumor-targeted PDT in 4T1-fLuc pulmonary metastatic mice. In the PDT group, lung metastasis was significantly limited, which supported the notion that the combined chemo-phototherapy and immunotherapy platform could effectively activate host antitumor immunity to prevent distant metastasis and inhibit breast tumor recurrence by stimulating immune memory [169]. The use of GBNs as a platform provides encouraging prospects for breast cancer treatment.

### Prospects

#### Targeted therapy

Targeted therapy is a treatment method at the cellular and molecular levels aimed at a well-defined carcinogenic site (the site can be a protein molecule or a gene fragment in the tumor cell) [206]. There are differences between targeted therapy and the abovementioned targeting effects. Here, the targeted ligands have the ability to not only specifically guide drugs to enter tumor cells but also inhibit the growth pathway of tumors to obtain therapeutic effects. Targeted therapy is currently the most promising therapeutic method for breast cancer. It can improve the bioavailability, biocompatibility, specific targeting and safety of therapeutic drugs [207]. However, these properties cannot be satisfied with single targeted therapy, so the combination of targeted therapy and chemotherapy is a current research hotspot. The strategy of coupling antibodies with chemotherapeutic drugs has been successfully developed. Antibody–drug conjugates such as T-DM1 and DS-8201 have been put on the market and yielded remarkable achievements in the treatment of breast cancer. Unlike the traditional method, the two treatments are not used separately but rather are loaded together into a graphene-based nanomaterial, which can increase their anticancer efficacy. GBNs can provide a platform for antibodies to bind potentially two or more chemotherapeutic drugs with different mechanisms. Herceptin, the most widely used targeted drug in the clinic, plays a profound role in HER2-positive breast cancer treatment [60]. Herceptin-conjugated GQDs loaded with DOX have been prepared for the treatment of HER2-positive breast cancer and can simultaneously deliver herceptin and DOX, taking advantage of the specific targeting effects of herceptin, and these GQDs exert a synergistically enhanced antitumor effect [127]. In addition to HER2 inhibitors, other types of targeted drugs, including CDK4/6 inhibitors, inhibitors of PARP, ER-targeting drugs, and PI3K/Akt/mTOR pathway inhibitors, are available. These new targeted drugs have achieved good results in clinical application and bring hope to breast cancer patients. Although there has been no relevant research, we believe that all of these new targeted drugs used with graphene-based carriers have the potential for multifold therapeutic effects. Future studies should focus on combining these various targeted drugs and chemotherapeutic drugs to find the appropriate ratio of antibody to chemotherapeutic drug to determine the best regimen. Furthermore, GBNs conjugated with targeted drugs are expected to overcome the problem of brain metastasis in breast cancer. The treatment of brain metastases in breast cancer remains difficult because herceptin and other macromolecular monoclonal antibodies have difficulty crossing the BBB [208]. Current studies have proven that GBNs can cross a variety of biological barriers, including the BBB [208]. The use of GBNs as carriers to assist these macromolecules in crossing the BBB can result in effective drug concentrations at intracranial tumor sites, bringing hope for the treatment of patients with brain metastases from breast cancer.

### Intratumoral drug delivery

Intratumoral drug delivery is an appealing locoregional therapy through direct intratumoral injection [209]. Compared with traditional intravenous injection, this strategy shows advantages in the loading and release of insoluble anticancer drugs. Intratumoral delivery sends chemotherapeutics directly to the breast tumor site, which reduces toxicity by avoiding exposure to normal tissues and improves the efficacy of the drugs [210]. The breast is a superficial organ, so it is suitable for intratumoral administration. There are two common types of intratumoral delivery systems for therapeutics: injectable gelling depots with thermosensitive hydrogels and preshaped implant systems. Injectable gelling depots are less invasive and less painful to inject. However, challenges with their intratumoral administration remain, including burst release, insufficient drug-loading capacity, implant biocompatibility and poor drug solubility issues that affect the release kinetics [211]. In addition, bolus injections into breast tumors can be quickly cleared, inspiring the exploration of drug-delivery carriers [212]. Graphene-based intratumoral drug-delivery systems could solve these limitations due to their controlled drug-release properties. Zhu et al. used chitosan gel-functionalized GO as an intratumoral delivery system to load docetaxel (DTX), which was showed a higher concentration and longer residence time in the breast tumor tissues of mice with no obvious toxicity to normal organs. This system proved to be a safe and effective intratumoral drug-delivery system for breast cancer treatment [197]. Fong et al. encapsulated FA-functionalized GO loaded with DOX into injectable hydrogels, which were administered by standard needles [46]. Intratumoral drug delivery has the advantages of low dose requirements, a reduced number of drug administration cycles and the absence of systemic drug uptake, which can minimize adverse effects [129]. Few intratumoral delivery formulations are currently available, and more graphene-based in situ intratumoral injectable formulations need to be explored. For example, suppose a subcutaneous, local, and intratumor drug-delivery system based on GBNs could be developed to maintain an effective drug concentration in the tumor in a continuous and controlled manner. New intratumoral delivery modes such as this could reduce patient hospitalization rates and length of stay, which would have economic benefits.

### Immunotherapy

**Fig. 7:**
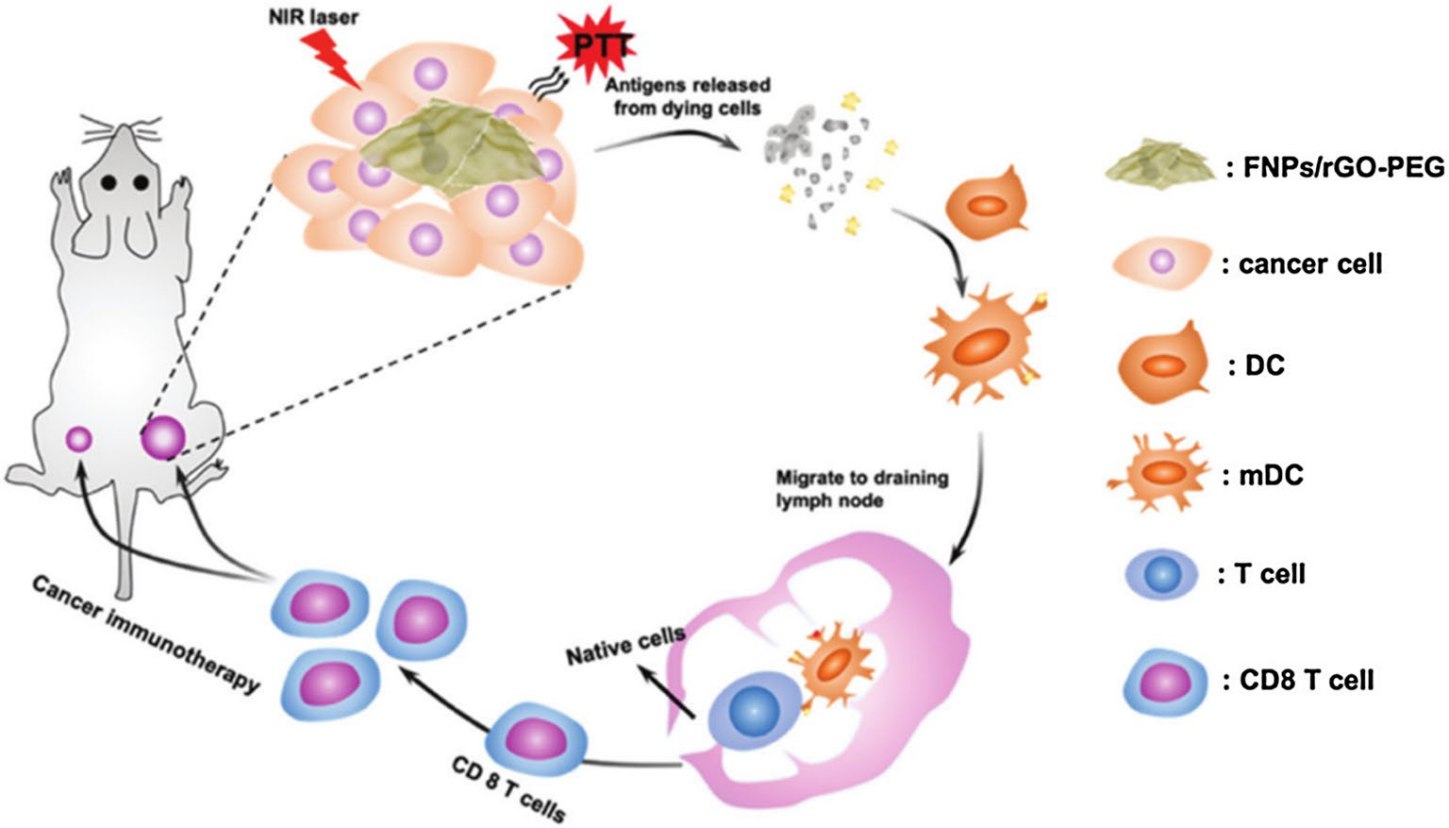
Schematic illustrations of NIR-mediated photothermal immunotherapy**.** A nanocomposite (FNPs/rGO-PEG) formulated by hybridizing Fe3O4 nanoparticles (FNPs), rGO and PEG-NH2 developed to destroy primary tumors was shown to elicit an anti-metastatic effect via photothermal immunotherapy. Under laser irradiation, FNPs/rGO-PEG nanocomposites generate heat and trigger immunogenic cell death (ICD). Then, the released tumor-associated antigens trigger the maturation of dendritic cells (DCs). DCs capture antigens and migrate to the tumor-draining lymph node to present antigens and activate T cells [166]. Reproduced by permission from the Royal Society of Chemistry

Tumor immunotherapy aims to activate the human immune system and kill cancer cells by autoimmune function. Unlike previous therapeutic strategies, the target of immunotherapy is not tumor cells but the body's own immune system. Immunotherapy can effectively remove residual cancer cells and small lesions after surgery, radiotherapy and chemotherapy; stop the regeneration of cancer cells; and prevent the recurrence and metastasis of tumors. Immunotherapy has the advantages of fewer side effects, increased biological sensitivity, and a persistent immune response [213]. GBNs can activate the immune system, creating opportunities for immunotherapy [214]. Yu et al. employed functionalized GO to trigger host immunity by activating dendritic cells and recruiting CD8^+^ T cell infiltration to enhance the immunological memory effect, which could inhibit the recurrence of breast cancer [169]. Recently, photoimmunotherapy has drawn increasing attention. PTT can produce tumor-associated antigens by causing immunogenic cell death, which leads to antitumor immunity. Wang et al. used hybridized Fe_3_O_4_ NPs and rGO nanocomposites to combine PTT and immunotherapy (Fig. 7), and the results showed that the primary breast tumor was destroyed through PTT and that distant metastasis could be prevented through the immune response activated by PTT-induced dendritic cell maturation and cytokine secretion [166]. Individualized treatment of tumors has always been a popular research topic. It is expected that a personalized cancer vaccine will be developed through the use of GBNs, which can be used as a carrier to deliver mRNA with patient-specific tumor antigen and protect it from degradation, inducing T cell antitumor immunity in the body. However, there have been few related studies on the application of GBNs in immunotherapy, and this appealing strategy calls for more attention.

## Conclusion

GBNs have the potential to be used in breast cancer diagnosis and treatment due to their distinctive structures and attractive physicochemical properties. To improve the application prospects of GBNs, balance between the anticancer ability and toxicity of GBNs is an urgent problem. Thus, we have summarized GBN modification strategies to enhance their targeting ability and biocompatibility. In addition, we stress the applications of GBNs in breast cancer treatment, including drug and gene delivery, phototherapy, and MTT, and as a platform to combine multiple therapies. Finally, we provide interesting perspectives on the potential applications of GBNs in targeted therapy, intratumoral drug delivery and immunotherapy. This review aims to provide information for drug design and clinical therapy, thereby facilitating the eventual application of GBNs in clinical breast cancer treatment.

## Abbreviations

AAP10: Antiarrhythmic peptide 10
ADR: Adriamycin drug resistance
Ag: Silver
A-Im: 1-(10-Bromoanthracene-9-yl)-1H-imidazole-4,5-dicarboxylic acid
AMF: Alternating magnetic field
Anti-MUC1 IgG: Mucin 1 receptor immunoglobulin G antibody
Apt-AuNP: Aptamer- gold nanoparticle
AptMUC1: MUC1-bindingaptamer
Au: Gold
AuNPs: Gold nanoparticles
CA IX: Carbonic anhydrase IX (CA IX)
CPPs: Cell penetrating peptide
CPT: Camptothecin
CuSQDs: Copper sulfide quantum dots
DESs: Deep eutectic solvents
DOX: Doxorubicin
DTX: Docetaxel
EA: Ellagic acid
EGFR: Epidermal growth factor receptor
EPR: Enhanced permeability and retention
ETS1: Erythroblastosis virus E26 oncogene homolog 1
F38: Pluronic F38
FA: Folic acid
FSHR: Follicle-stimulating hormone receptor
FU: Fluorouracil
GA: Gambogic acid
GBNs: Graphene-based nanomaterials
Gd: Gadolinium
GDC0941: A phosphatidylinositide 3-kinase/Akt phosphorylation inhibitor
Gd-DTPA: Gd diethylene penta-acetic acid
GO: Graphene oxide
GPD: Hybrid vector consists of GO: PAMAM and PEG
GQDs: And graphene quantum dots
GSH: Glutathione
Hap: Hydroxyapatite
HER2: Human epidermal growth factor receptor 2
HIF: Hypoxia-inducible factor
hMSN: Hollow mesoporous silica nanoparticles
HPPH: A photosensitizer
HSP: Heat shock protein
HSV-TK: Herpes simplex virus thymidine kinase gene
HSV-TK: Herpes simplex virus thymidine kinase gene
HY: Hypericin
IC50: The half maximal inhibitory concentration
IONP: Iron oxide nanoparticle
L-Arg: L-arginine
MB: Methylene blue
MD: Maltodextrin
MDR: Multidrug resistance
MDR1: Multidrug resistance 1
miR-101: MicroRNA-101
MIT: Mitoxantrone
MRI: Magnetic resonance imaging
MTD: Magnetothermodynamic
MTT: Magnetothermal therapy
MTX: Methotrexate
NIR: Near infrared
OCT: Octreotide
PA: Photoacoustic
PAH: Poly(allylamine hydrochloride)
PAMAM: Poly amidoamine
pDA: Polydopamine
PDT: Photodynamic therapy
PEG: Polyethylene glycol
PEG-diamine: Aminated-polyethylenglycole
PEI: Polyethylenimine
PET: Positron emission tomography
PF-127: An amphiphilic polymer
P-gp: P- glycoprotein
P-L-Arg: Poly-L-arginine
PL-PEG: A phospholipid-based amphiphilic polymer
Pt: Platinum
PTT: Photothermal therapy
PTT: Photothermal therapy
PTX: Paclitaxel
PVP: Polyvinylpyrrolidone
R8: Octaarginine
rGO: Reduced graphene oxide
ROS: Reactive oxygen species
siRNA: Small interfering RNA
SPECT: Single-photon emission computerized tomography
T80: Tween 80
Tf: Transferrin
TPGS: D-α-tocopheryl polyethylene glycol 1000 succinate
β-CD: β- Cyclodextrin
